# Adsorption of Uranium, Mercury, and Rare Earth Elements from Aqueous Solutions onto Magnetic Chitosan Adsorbents: A Review

**DOI:** 10.3390/polym13183137

**Published:** 2021-09-16

**Authors:** Georgia Michailidou, Ioanna Koumentakou, Efstathios V. Liakos, Maria Lazaridou, Dimitra A. Lambropoulou, Dimitrios N. Bikiaris, George Z. Kyzas

**Affiliations:** 1 Laboratory of Polymer Chemistry and Technology, Department of Chemistry, Aristotle University of Thessaloniki, 54124 Thessaloniki, Greece; michailidougeorgia18@gmail.com (G.M.); iwanna.koumentakou@gmail.com (I.K.); marlazach@chem.auth.gr (M.L.); 2 Department of Chemistry, International Hellenic University, 65404 Kavala, Greece; stathilas@gmail.com; 3 Laboratory of Environmental Pollution Control, Department of Chemistry, Aristotle University of Thessaloniki, 54124 Thessaloniki, Greece; dlambro@chem.auth.gr

**Keywords:** chitosan, synthesis, characterization, pH, isotherms, adsorption capacity, kinetics

## Abstract

The compound of chitin is the second most important and abundant natural biopolymer in the world. The main extraction and exploitation sources of this natural polysaccharide polymer are mainly crustaceans species, such as shrimps and crabs. Chitosan (CS) (poly-β-(1 → 4)-2-amino-2-deoxy-d-glucose) can be derived from chitin and can be mentioned as a compound that has high value-added applications due to its wide variety of uses, including pharmaceutical, biomedical, and cosmetics applications, food etc. Furthermore, chitosan is a biopolymer that can be used for adsorption applications because it contains amino and hydroxyl groups in its chemical structure (molecules), resulting in possible interactions of adsorption between chitosan and pollutants (uranium, mercury, rare earth elements (REEs), phenols, etc.). However, adsorption is a very effective, fast, simple, and low-cost process. This review article places emphasis on recent demonstrated research papers (2014–2020) where the chemical modifications of CS are explained briefly (grafting, cross-linking etc.) for the uptake of uranium, mercury, and REEs in synthesized aqueous solutions. Finally, figures and tables from selected synthetic routes of CS are presented and the effects of pH and the best mathematical fitting of isotherm and kinetic equations are discussed. In addition, the adsorption mechanisms are discussed.

## 1. Introduction

Chitosan (CS) is a natural linear polysaccharide composed of (1,4)-2-amino-2-deoxy-b-D-glucan, and can be fabricated by partial chitin deacetylation [1]. CS has received great attention due to numerous amine and hydroxyl functional groups in its structure. CS possesses excellent properties, such as a low price and abundance, antibacterial capacity, low toxicity, biodegradability, biocompatibility, hydrophilicity, and high adsorbent ability [2]. The dissolution of CS in aqueous acids provokes the protonation of the amino groups along the chitosan, creating cationic sites. This unique property increases the solubility, polarity, and ability of CS to adsorb different pollutants. For example, protonated amine groups can attract metal anions [3]. Several studies have focused on the elimination of different wastes from aqueous solution using CS-based adsorbents [2].

Water is the most essential component for all living organisms on earth. However, owing to the tremendous growth of the planet’s population, along with industrialization and technology development, water pollution issues arise [4]. Heavy metals, including mercury, uranium, cobalt, copper, zinc, chromium, cadmium, lead, nickel, arsenic etc., are usually inserted in lakes, reservoirs, groundwater, streams, and rivers, mainly due to industrial effluents [5]. They are toxic, non-biodegradable, and exist in different oxidation states for long periods in the environment. Since these metals are found in water streams, human exposure to them is inevitable. When entering the human body, metals accumulate in certain selected tissues and may become toxic and harmful [6]. The World Health Organization estimates that, by 2030, billions of people will lack access to safe water, sanitation, and hygiene. 

Over the years, different methods have been developed for the separation of heavy metals, such as chemical precipitation, ion exchange, nanofiltration, reverse osmosis, solvent extraction, and sorption. Among these methods, sorption is the most financially feasible method, especially for the treatment of dilute metal solution materials [2,7], attributed to its ease of application and high effectiveness without further pollution of the environment. Activated carbon has been vastly applied as an effective adsorbent. Nevertheless, the high cost of its thermal regeneration has turned the research interest to other effective adsorbents, including CS nanoparticles [8]. Moreover, the preparation of magnetic particles renders the consequent separation effortless with an external magnetic field. 

Thus, the present review focuses on uranium (U), mercury (Hg), and rare earth elements (REEs) removal with CS magnetic particles over the years 2014–2020. CS chemical modifications for a more efficient uptake of uranium, mercury, and REES are described, while the impact of pH and the optimum mathematical fitting of isotherm and kinetic equations to the adsorption mechanisms are presented. 

## 2. Synthetic Routes and Characterizations

### 2.1. Synthetic Routes and Characterizations for Uranium Adsorption

Uranium (U) is a representative actinide metal element with atomic number 92. It is a strategic resource in the nuclear industry and, as a result, uranium plays an important and irreplaceable role in the production of nuclear energy [9]. However, it is chemically and radioactively toxic, and has a long half-life with a high radiological and biological toxicity, causing irreversible damage in humans and the environmental ecosystem [10,11]. Inevitably, uranium can escape into the environment in the mining processes, nuclear research, and weapons manufacture, as well as due to improper nuclear waste management, and nuclear safety accidents [9]. As a result, the research associated with uranium removal from seawater and nuclear industrial wastewater has gained a lot of attention [12]. 

During the last several years, many magnetic CS composite systems have been developed, such as the magnetic hydrothermal cross-linked chitosan (HCC-Fe_3_O_4_), in order to synthesize a low-cost and highly efficient adsorbent with a high adsorption capacity. The successful synthesis of HCC-Fe_3_O_4_ was confirmed from FT-IR patterns, where characteristic peaks of CS and Fe_3_O_4_ (Fe-O bond at 592 cm^−1^) were present. Scanning electron microscopy (SEM) images present differences in the morphology of structures with and without loaded Fe_3_O_4_ nanoparticles. HCC showed a disordered fiber structure, whereas the surface of HCC-Fe_3_O_4_ was smooth and uneven. The magnetic properties of HCC-Fe_3_O_4_ were proved with the use of a magnet. The results showed that HCC-Fe_3_O_4_ dispersed in a water solution could be easily separated from water with a magnet, in contrast with HCC/water, which was still cloudy [10].

**Figure 1:**
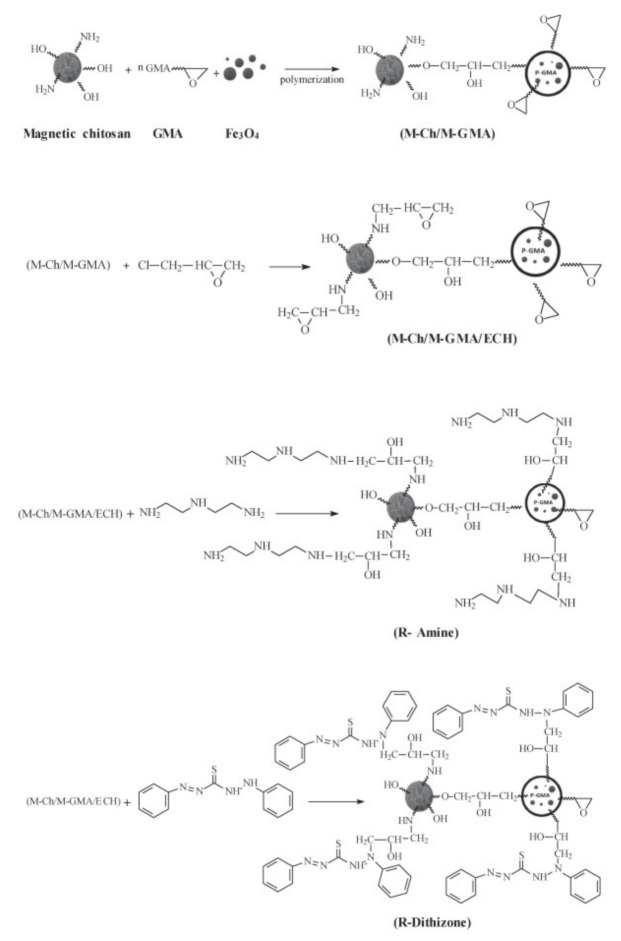
Synthesis routes for the preparation of R-Amine and R-Dithizone sorbents. Reprinted from Ref [13]. Copyright 2021 with permission from Elsevier B.V.

Another interesting property of CS is its readiness to be chemically modified due to the reactivity of its hydroxyl and amine groups. The chemical modification of magnetic chitosan microparticles with new functional groups combines fast sorption processes with a high reactivity and selectivity. Uranium has a great affinity for amine, phosphonic, carboxyl, and amidoxime groups. For this purpose, magnetic/CS nanoparticles decorated with glycidyl methacrylate (GMA) were functionalized with amine groups (diethylenetriamine, DETA). The standard support was also functionalized using dithizone (Figure 1). SEM and transmittance electron microscopy (TEM) figures of R-Amine and R-Dithizone showed irregular surfaces, presenting a combination of micro-spherical objects, plane plates, and aggregates. The images of the magnetic particles (magnetite-embedded chitosan functionalized with GMA) of R-Amine and R-Dithizone showed unexpected differences, taking into consideration that they should be the same. Magnetic particles of R-Amine presented an irregular shape with a particle size ranging between 8–19 nm and 6–13 nm. R-Dithizone showed small irregular particles (less than 10 nm) and spherical structures (ranging between 40 and 120 nm). The coating layer of magnetite nanoparticles, which is constituted of non-magnetic material, contributes to the reduction of the magnetization properties of the material (shielding effect). However, the aforementioned decrease affects the separation ability of the magnetic nanoparticles under an external magnetic field. The thermal degradation of R-Amine and R-Dithizone are relatively close, exhibiting that the residual weights after complete degradation are close to 15 and 11%, respectively. However, DTG curves showed substantial differences between the two sorbents, proving that the water uptake of R-Dithizone was higher in comparison to R-Amine (diphenylthiocarbazone for producing GMA-magnetic/CS-dithizone NPs, R-Dithizone, for comparison with R-Amine sorbent) [13].

Furthermore, magnetic CS nanoparticles were modified with humic acid via interactions of the various active moieties of humic acid (carboxylic, hydroxyl, phenolic, amino groups) with CS by bridging Fe_3_O_4_. The spherical structure of the nanocomposite powder was visible from SEM images [14]. As aforementioned, phosphate groups and carboxyl groups have excellent chelating properties for uranium removal. 2-phosphonobutane-1,2,4-tri carboxylic acid (PBTCA) was grafted onto CS-coated magnetic silica nanoparticles. PBTCA contains both carboxyl and phosphonic groups in one molecule, which can improve uranium coordination. PBTCA was successfully grafted onto nanoparticles via the amidation reaction between the amino groups of CS and the carboxyl groups of the PBTCA molecule. The crystal structure of magnetic CS nanoparticles was not destroyed during silica coating, CS coating, and PBTCA grafting [9]. In addition, Zhou et al. prepared triphosphate-crosslinked magnetic CS resins. The semi-quantitative analysis from the energy-dispersive X-ray spectroscopy revealed an atomic percentage of phosphorus in the new sorbent, indicating that CS was successfully cross-linked by sodium tripolyphosphate (TPP) and attached to the surface of magnetic particles. FT-IR spectra presented vibrations attributed to the TPP molecule (P=O stretching vibration at 1253 cm^−1^, P-O of the P-OH groups at 1640). All of the characteristic peaks of CS related to N atoms were affected after the cross-linking process, indicating that cross-linking was developed by the ionic interaction between -NH_3_ and -O-P groups. The magnetic property of composites was confirmed from the SEM micrograph, which showed small particles (200–350 nm) that were easily separated from the water solution, within 1–2 min, by applying a magnetic field [1].

On the other hand, the modification of CS can cause the limitation of its available amine groups, decreasing their reactivity for metal complexation. The scientific community attempted to reduce this phenomenon by cross-linking magnetic CS nanoparticles with epichlorohydrin. Specifically, Mohammad G. et al. prepared cross-linked magnetic CS nanoparticles with epichlorohydrin and then grafted diethylenetriamine onto them. The particles presented a homogeneous spherical morphology. They formed agglomerates due to the strong magnetic dipole–dipole attraction between particles. This can be explained by the difference in the electron-absorbing ability of the magnetic iron core and CS shell compartments. The size of nano-based magnetic particles was hardly affected by CS. Magnetic CS nanoparticles showed superparamagnetic abilities, proving their easy separation with the help of an external magnetic field [15]. Similarly, magnetic CS microparticles, after being activated with epichlorohydrin, were grafted with malononitrile. In the last step, amidoximation of the sorbent was prepared. The CS coating represented approximately 60–64% of the total weight of the composite, affecting its crystallinity [16]. 

Magnetic CS nanocomposites with core-shell structures (the magnetic core was coated by the CS shell) were also cross-linked with epichlorohydrin via the hydrothermal precipitation method, and were then modified with polyethyleneimine (PEI). TEM images showed that the magnetic nanoparticles were uniformly dispersed within the polymer matrix. Fe_3_O_4_ nanoparticles were encapsulated in CS, increasing the diameter of the particles. The presence of CS and the grafting of PEI did not disrupt the crystallinity of the magnetic nanoparticles. On the opposite side, the addition of PEI in the CS shell reduced the magnetization [17]. 

In another study, magnetic CS particles were crosslinked with epichlorohydrin and were then chemically modified with triethylenetetramine. FT-IR spectrums confirmed the successful chemical modification of the polymeric matrix and the increase in functional amine groups, which, in turn, represent the active sites on the adsorbent. The X-ray diffraction patterns recorded that, after chemical modification, the weak crystallinity of the biopolymer was drastically reduced, and the polymer became amorphous [18]. A new kind of hydrazide derivative of CS (supported on magnetic nanoparticles) was designed in five steps. FTIR spectra proved the efficient synthesis of the sorbent at the different steps: (i) synthesis of magnetite/CS particles (broad peak in the range of 565–550 cm^−1^), (ii) activation of the composite material through epichlorohydrin grafting (at around 806 cm^−1^), (iii) grafting of nitrile functions (the peak at 2360–2330 cm^−1^), (iv) hydrazinyl amination (around 1724 cm^−1^), and (v) efficient substitution of hydrazide. Energy dispersive X-ray analysis (EDX) and elemental analyses also confirmed the effective composition of the material, showing that the grafting of a new reactive group is highly efficient. The semiquantitative analysis of the sorbent before and after the metal ion presented a satisfying amount of Fe in the magnetite core (close to 40%, *w*/*w*). Additionally, O was detected at the polymeric coating (30% *w*/*w*) and C and N at the surface of the magnetite microparticles. TEM images presented dense magnetite particles of approximately 10 nm with an irregular shape. The sorbent showed a relatively low specific surface area, meaning that the material presented a low porosity [19].

Additionally, financially friendly, as well as environmentally friendly sorbents, were prepared for the removal of U(VI). Magnetic-Momordica charantia leaf powder was impregnated into CS (m-MCLPICS) to produce a sorbent with a good U(VI) sorption capacity. The impregnation of the material increased the surface area, pore volume, and diameter, whereas it reduced the Fe_3_O_4_ content, which will further decrease the magnetization. SEM images demonstrated a smooth surface with plates and a nearly regular surface before the sorption of U(VI), whereas, after sorption, the surface was completely altered. A granular substance on the adsorbent surface was observed, which proves the existence of the adherent of U(VI) ions on the surface [11].

**Figure 2:**
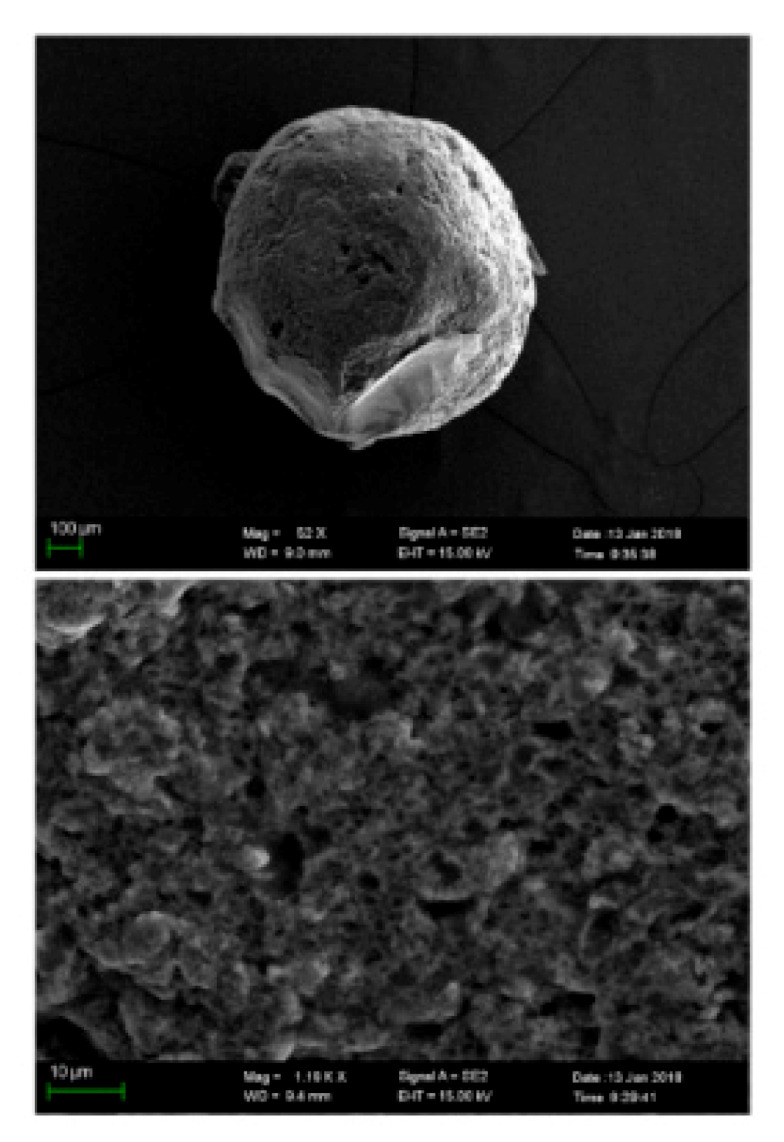
SEM images of magnetic amidoxime chitosan beads. Reprinted from Ref [12]. Copyright 2018 with permission from Elsevier Ltd.

Another environmentally friendly U(VI) adsorption material was prepared, synthesizing magnetic amidoxime CS beads. Firstly, -CN groups were grafted onto the CS backbone, and then the conversion of -CN groups into amidoxime groups was carried out. SEM images confirmed the spherical structure of the CS bead with a diameter of around 1 mm and a porous surface (Figure 2) [12]. CS-carboxymethyl cellulose magnetic microspheres were modified with phytic acid, which is a kind of natural organic phosphoric compound, via a self-assembly process. The composites presented excellent superparamagnetic and superhydrophilic properties. The modification with phytic acid did not affect the crystallization, but decreased the magnetization of the materials. Naked Fe_3_O_4_ particles showed uniform spheres with a smooth surface. After CS modification, the microspheres maintained the spherical shape. However, a smaller cluster, including small nanoparticles, was formed. This was observed due to the fact that CS expelled the captured NH_3_ bubbles and stabilized the formation of magnetic substrates in the reaction [20].

### 2.2. Synthetic Routes and Characterizations for Mercury Adsorption

Mercury (Hg) is a transition metal with atomic number 80. It is a heavy, d-block metallic element, with the weakest metallic bonding among the other metals and a melting point of −39 °C, rendering it as a liquid at standard conditions for temperature and pressure. Hg^2+^ ions are considered as extremely toxic, nonbiodegradable, bioaccumulated pollutants with long-term residence at low concentrations in water, causing a series of human disorders, such as kidney failure or cardiovascular system destruction [21]. Every year, thousands of tons of Hg are released into the environment from natural and anthropological sources [22]. Consequently, owing to the large amount of Hg in wastewaters, along with its high toxicity, its removal from aqueous solutions is rendered obligatory.

Over the years, many common strategies for the effective removal of Hg ions have been examined. The formation of CS beads containing a ferrous core is a common technique for their removal. EDX analysis confirms the successful inclusion of Fe^3+^ ions in the CS nanoparticles, leaving their inner structure unaffected. The resulting particles are spherically shaped with a rough surface and inner porosity. Thermal analysis of the neat CS and CS nanocomposite adsorbent reveals the enhanced thermal stability of the final beads, which is attributed to the presence of an iron core, where the conversion of the iron(III) inorganic core into magnetite, maghemite, or wüstite Fe_1−x_O at a temperature of over 600 °C takes place [23].

In a further step, the sorption of Hg^2+^ with magnetic nanoparticles has been applied. Magnetic nanoparticles are mostly prepared through a chemical precipitation method according to the Equation (1), whereas, thereafter, CS nanoparticles containing a magnetic core are prepared via an ionic gelation technique. The magnetic beads are nanoscaled spherical dots, and the application of a coating layer results in an increase in their size and the formation of rougher surfaces.

2FeCl_3_ + FeCl_2_ + 8NH_3_ + 4H_2_O → Fe_3_O_4_ + 8NH_4_Cl (1)

The employment of various materials as coating layers has been extensively applied. Polythiophene was applied as a coated material onto the CS magnetite nanocomposite. Concerning its crystalline structure, it remained unaffected throughout the preparation of the nanocomposite [24]. A P-sulfonato dansyl calixarene coating layer was applied onto a CS magnetic core. Owing to the presence of opposite charged ions, self-assembly occurs and colloidal polyion complexes are formed. The coating of the magnetic core with an oppositely charged bilayer results in aggregated sphere-shaped particles, while the highly crystalline magnetic core is uniformly distributed in the polymeric matrix [21].

**Figure 3:**
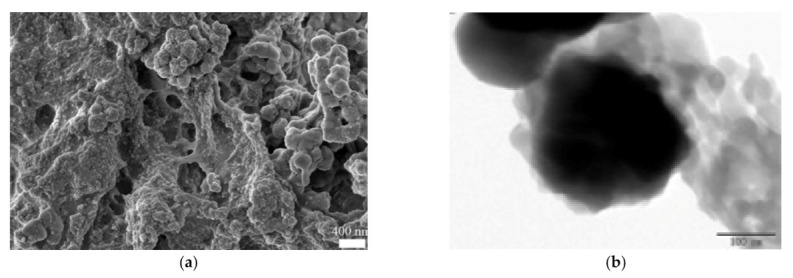
(**a**) SEM images and (**b**) TEM images of CS- and poly(m-aminothiophenol)-coated magnetic nanocomposite. Reprinted from Ref [25]. Copyright 2020 with permission from Elsevier B.V.

Furthermore, a magnetic network polymer composite material composed of CS and poly(m-aminothiophenol) has been prepared with tannic acid as a cross-linker. The sulfur and nitrogen groups of the final adsorbent are able to form strong coordination bonds with Hg ions. Concerning its crystalline state, the magnetic core was not affected by the procedures applied for the preparation of the adsorbent. The polymer coating was wrapped around the black magnetic nanoparticles and cross-linked pores were randomly distributed, attached on the surface of the magnetic carrier (Figure 3) [25].

Moreover, ethylenediaminetetraacetic-disodium (EDTS-Na_2_) was modified onto magnetic CS particles. Zeta potential is an important factor affecting the adsorption procedure, defined as the potential that exists at the boundaries of the outer diffuse layer surrounding charged particles. Consequently, EDTA-Na_2_ was immobilized on the Fe_3_O_4_ core; since CS formed a nanosphere around the magnetic core, the presence of EDTA-Na_2_ did not significantly affect the ζ potential values of the sorbents [22]. Concerning the magnetic properties, pure Fe_3_O_4_ nanoparticles present a super-paramagnetic character while the coating with a CS layer affects the final magnetization. Further coating layers drastically affect the saturation magnetization of the sorbent. Coating the magnetic core with a layer of p-sulfonato dansyl calix(4)arene does not affect the saturation magnetization value, resulting in a superparamagnetic material, whereas coating the magnetic core with polythiophene, poly(m-aminothiophenol), or EDTA-Na_2_ results in lower saturation magnetization values, attributed to the shielding of the magnetic core from the polymeric material [21].

Many research groups have focused on Hg removal through magnetic nanocomposite adsorbents of functionalized CS. Thiol-functionalized CS magnetic nanoparticles were prepared through a capillary microfluidic device, based on an ionic gelation technique. The CS derivative was prepared with epichlorohydrin and cysteaminium chloride, and their initial in-between molar ratio utilized at the functionalization of CS is crucial and affects the imminent successful removal of Hg ions. The effectiveness of the final magnetic particles is affected by their degree of crystallinity, since a higher degree of crystallinity results in a lower efficacy of the sorbent during the adsorption procedure [26].

**Figure 4:**
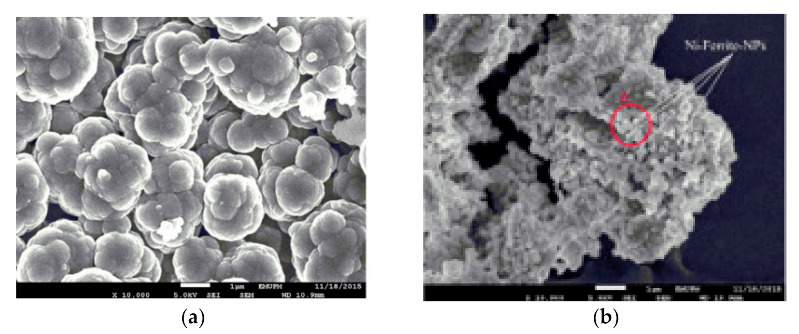
FE-SEM image of (**a**) neat polypyrrole-CS nanoparticles and (**b**) polypyrrole-CS/nickel ferrite (NiFe_2_O_4_) composite nanoparticles. Reprinted from Ref [29]. Copyright 2017 with permission from Elsevier Ltd.

In another study, aminothiourea functionalized CS was covalently grafted onto NH_2_-Fe_3_O_4_ nanoparticles, with glutaraldehyde as the crosslinker. The magnetic core is thermally stable and thermogravimetric analysis (TGA) confirms that, between the temperature range 200–520 °C, the mass loss is solely attributed to the modified CS. Concerning their obtained size distribution, initial NH_2_-Fe_3_O_4_ particles were monodispersed, while the addition of modified CS enlarged the size of the nanocomposite [27]. The size of the obtained nanocomposites was examined in a comparison between CS and glutamine modified CS magnetic silica coated core composite microspheres. The silica coating layer was applied for the improvement of the acid resistance of the particles. All of the obtained particles are of a spherical shape, but the CS-modified spheres tend to have a larger size and consequently a larger surface area and, hence, a greater expected adsorption capacity [28]. In addition to the aforementioned, agglomeration was observed in the polypyrrole-CS/nickel ferrite (NiFe_2_O_4_) composite material. The nanoparticles were prepared under the electrochemical polymerization of pyrrole in the presence of NiFe_2_O_4_. The neat polypyrrole-CS nanoparticles were agglomerated; however, the addition of the magnetic NiFe_2_O_4_ nanoparticles further increased its agglomeration, affecting the morphology of the final product (Figure 4) [29]. Regarding the saturation magnetization of the final nanocomposite materials, in any case, the inclusion of the magnetic core in the modified CS matrix diminishes the saturation magnetization value of the sorbent. It is interesting to note the silica-coated glutamine modified CS nanoparticles. The presence of the silica coating reduces the contents of Fe_3_O_4_ nanoparticles in the final adsorbents; thereafter, a reduction of the saturation magnetization value is expected. However, all of the aforementioned beads are able to be separated magnetically under an external magnetic field.

### 2.3. Synthetic Routes and Characterizations for Rare Earth Elements Adsorption

Rare earth elements (REEs) are a group of chemical elements that include 17 metals, i.e., lanthanum (La), cerium (Ce), praseodymium (Pr), neodymium (Nd), promethium (Pm), samarium (Sm), europium (Eu), gadolinium (Gd), terbium (Tb), dysprosium (Dy), holmium (Ho), erbium (Er), thulium (Tm), ytterbium (Yb), and lutetium (Lu) [30]. Over the years, lanthanides have been utilized in many fields of applications, including catalysts systems and wireless technologies, as well as in nuclear, medical, and dental areas, as well as lasers, fertilizers, superconductors, and optical fibers. Lanthanides are considered as non-toxic materials because they are unable to pass through the cell membranes. However, when injected intravenously, they are able to pass through calcium ions and consequently be poisonous [31]. 

Many separation techniques have been applied during the last several years for the effective removal of metals, dyes, and organic compounds from wastewaters. Among the adsorbents, magnetic separation techniques are known to be quick and easy methods for the sensitive and reliable capture of the aforementioned pollutants [32]. Magnetic nanoparticles combine a large specific surface area, leading to an excellent adsorption performance along with the feasibility of their separation. Coating magnetic Fe_3_O_4_ nanoparticles with CS results in a porous adsorbent that is capable of adsorbing and desorbing REEs. The presence of free amino groups of the CS structure renders the adsorption more effective at pH 3, since, in an acidic environment, the amino groups are protonated and therefore suitable for adsorption [31]. The conditions applied for the preparation of the magnetic nanoparticles drastically affects their saturation magnetization. Manganese ferrite MnFe_2_O_4_ nanoparticles prepared under three different temperatures (60, 70, 80 °C) with the co-precipitation technique were examined, and the particles with the most significant saturation magnetization, along with the optimum crystallite size, were found to be the particles prepared at 60 °C. Further coating of the nanoparticles results in a hybrid composite material with a rough surface and cracked features. The presence of CS results in the diminishing of the saturation magnetization of the hybrid particles; in comparison to uncoated MnFe_2_O_4_ nanoparticles, however, the resulting beads are able to be separated by magnetic methods [33]. The preparation of the CS/magnetite composite materials is developed mainly through the coprecipitation method, where iron (II) and iron (III) salts are precipitated by aqueous ammonia in the CS solution. The presence of CS does not affect the final crystal structure of the composite material. However, its specific surface area is diminished in comparison to unmodified magnetite, along with the pore size, since CS tends to partially fill the pores, forcing their redistribution between particles [34]. The final morphology of the hybrid particles’ surface facilitates their entrance into the aqueous phase and the adsorption of Nd^3+^ ions into the hybrid beads [33].

**Figure 5:**
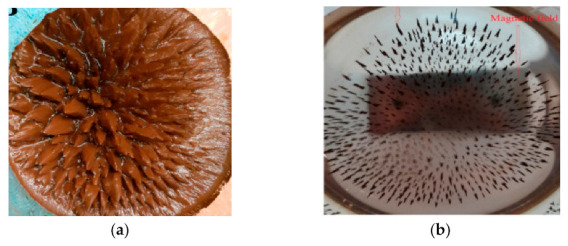
Photo of Ni_0.2_Zn_0.2_Fe_2.6_O_4_ nanoparticles prepared from hydrothermal procedure (**a**) before drying and (**b**) in solution under magnetic field after drying. Reprinted from Ref [35]. Copyright 2020 with permission from Elsevier B.V.

For the successful formulation of CS magnetite composite sorbents, alginate and CS have been utilized together. Since these polysaccharides are oppositely charged, with carboxylic and amino groups, respectively, they are able to interact through electrostatic interactions and successfully form beads while enclosing a magnetic core in their interior. The resulting magnetic beads are dark and spherical, with the ability to be easily removed from the aqueous solution by an external magnet (Figure 5) [32].

A bionanocomposite composed of calcium alginate, carboxymethyl CS, and Ni_0.2_Zn_0.2_Fe_2.6_O_4_ was prepared for the magnetic adsorption of REEs. Ni_0.2_Zn_0.2_Fe_2.6_O_4_ nanoparticles are synthesized through a hydrothermal technique. The final particles are almost spherical in shape, homogenously sized, and are distributed with nano-sized pores. They are superparamagnetic, with a saturation magnetization value equal to 45.87 emu/g. The formation of the composed nanoparticles affects their thermal degradation behavior. TGA analysis reveals three degradation steps attributed to bound water and to the degradation and decomposition of the polymeric matrix. The final residue percentage is ascribed to the presence of the magnetic core [35]. The effect of the gelation of the aforementioned nanoparticles with CaCl_2_ and glutaraldehyde, as well as the effect of the presence of pyrimidine-thiophene-amide onto the calcium alginate/carboxymethyl CS/Ni_0.2_Zn_0.2_Fe_2.6_O_4_ composite nanoparticles, were assessed [36,37].

**Figure 6:**
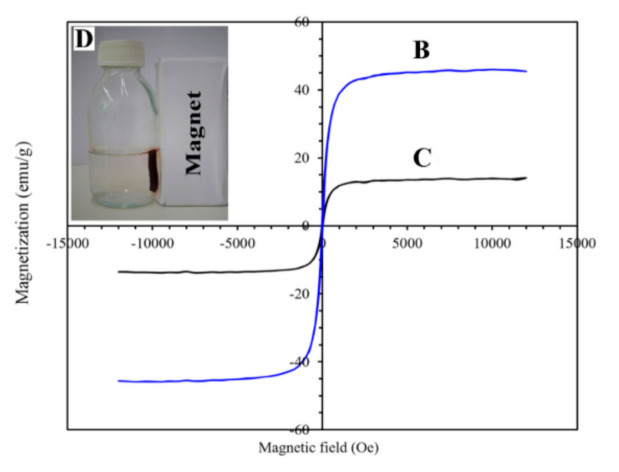
Saturation magnetization curves of (B) Ni_0.2_Zn_0.2_Fe_2.6_O_4_ and (C) CA/CMC/Ni_0.2_Zn_0.2_Fe_2.6_O_4_; (D) magnetic separation of the ions-loaded adsorbent. Reprinted from Ref [35]. Copyright 2020 with permission from Elsevier B.V.

In any case, the prepared bionanocomposite material has a lower saturation magnetization value, owing to the interaction of the magnetic nanoparticles with alginate carboxymethyl CS and poly(pyrimidine-thiophene-amide). However, the low values are sufficient for a successful separation of the magnetic beads under an external magnetic field, as depicted in Figure 6.

The application of CS derivatives onto magnetic nanocomposite adsorbents has been examined by many research groups. Pure Fe_3_O_4_ is a highly porous material, with a large specific surface area, and its nanoparticles tend to aggregate, owing to the strong magnetic dipole-dipole interactions. Coating its surface with a thin layer of modified diethylenetriamine CS (FCCD) affects its porosity, as well as the hydrophilicity of the FCCD [38]. Consequently, grafting CS onto the magnetic nanoparticles’ surface results in the diminishing of the specific surface area of the final hybrid material. The group of Pylypchuk et al. specifically examined the effect of the modification of a magnetite on its specific surface area. The (3-Aminopropyl) triethoxysilane (APTES) modified magnetite surface was smooth, with a high specific surface area value. CS was grafted onto the modified magnetic nanoparticles’ surface, which led to the reduction of their specific surface area. However, the further modification of the immobilized CS structure with diethylenetriaminepentaacetic acid (DTPA) resulted in the further addition of carboxylic groups on the hybrid material’s surface and the additional augmentation of the specific surface of the final hybrid beads [39].

Apart from the aforementioned, the effect of grafting molecules onto the magnetic core on the thermal and crystalline behavior is evaluated. The grafting of malonitrile onto the magnetic CS nanoparticles, as well as the consequent amidoximation of the sorbent, were assessed for the adsorption of Eu from aqueous solutions. Magnetite nanoparticles prepared by the hydrothermal co-precipitation method are highly crystalline, whereas their crystallinity is reduced when enclosed into the polymeric semi-crystalline CS modified matrix. However, the position of the crystalline peaks is constant, confirming the synthesis of a magnetic core inside the particles [16]. Moreover, the crystalline behavior of functionalized poly(aminocarboxymethylation) CS (PCM-CS) containing a magnetic core was assessed. The final composite material reveals a lower degree of crystallinity in comparison to neat CS, as well as lower values of magnetization when compared to neat Fe_3_O_4_ [40].

Concerning the saturation magnetization values of the hybrid materials, in any case, they are slightly lower than the neat magnetic core, confirming the successful coating or grafting of the nanoparticles. The magnetization is proportional to the amount of magnetite particles added. Nevertheless, the final hybrid materials are able to adsorb REEs in their interconnected structure and respond to the external magnetic field for the separation of the sorbent with the pollutant.

## 3. Adsorption Evaluation

### 3.1. Isotherm Models and Kinetic Equations

In this review paper, regarding the use of composite nanoadsorbents, some theoretical equations (models) are presented, explained, analyzed, and some crucial parameters of the adsorption process, such as the adsorption capacity and kinetic rate, are evaluated.

#### 3.1.1. Isotherm Models

The analysis of innovative adsorbents presupposes the selection of the most suitable adsorption equilibrium correlation in order to select the optimal system of adsorption. The isotherms (equilibrium equations) derived from the adsorption process (experimental data), are essential for the optimization of adsorption mechanism paths, the expression of surface properties and adsorption capacities of synthesized nanoadsorbents, and also the model design of the adsorption systems, in order to explain how to interrelate the selected adsorbent materials with the model pollutants. The explanation of the aforementioned phenomenon can be attributed to the release or mobility of a (selected) substance from the aqueous porous media or aquatic environments to a solid-phase (composite nanoadsorbent) at a persistent pH and temperature, in broad-spectrum, giving an invaluable curve. The plotting graph derived from the solid-phase and model pollutant concentration in general reveals the mathematical association and/or fitting towards the operational design, modeling analysis, and the applicable practice of the (selected) adsorption systems. 

In the case where the concentration of the solute solution in the aqueous solution remains unchanged and/or stable, a fact that is attributed to a zero net transfer of solute adsorbed and then desorbed from the surface of the synthesized composite nanoadsorbent (solid-phase), the equilibrium phase is obtained. However, the equilibrium concentration that presents the selected model pollutant (adsorbate) in the solid and liquid phase, at a predefined temperature, can be indicated by the isotherms of sorption derived from the phase of equilibrium. The shapes of derived isotherms that can be obtained are divided into linear, unfavorable, favorable, strongly favorable, and irreversible types. In addition, a wide variety of equilibrium isotherm models have been developed in the past (Table 1) [41]. The amount of model pollutants that were taken from the aqueous solutions and achieved the equilibrium phase Q_e_ (mg/g) can be calculated according to the mathematical Equation (2) of mass balance and is given by:

(2) $$Q_{e}=\frac{\left(C_{0}-C_{e}\right)V}{m}$$

where C_0_ and C_e_ (mg/L) are the initial and equilibrium concentration of the model pollutants, respectively; V (L) and m (g) are the volume of adsorbate (solution) and the mass of adsorbent, respectively.

#### 3.1.2. Kinetic Equations

The prediction of the optimal condition of the processes is very important, and is based on the results that are obtained from the kinetic studies. The kinetic modeling can provide details on the mechanism, which contributes to the phase of adsorption and possible rate-controlling steps, such as the processes of mass transport or chemical reaction. However, over the years, research has developed several kinetics models, but it must be mentioned that the most prevalent models are the pseudo-first order and pseudo-second order equations. Moreover, other kinetic models, such as Elovich and intra-particle diffusion, are not extensively used [41]. In Table 2, the aforementioned kinetic models forms are presented.

### 3.2. Discussion

Below, several selected studies for the uptake of uranium (Table 3) from synthetic aqueous solutions, by using composite adsorbents derived from chitosan, are presented. In addition, the pH (optimum), preferred, and best mathematical fitting of isotherms models and kinetics equations, and also the adsorption capacity (Q_max_) of the selected composite adsorbents derived from chitosan are presented. The isotherm models used are Langmuir (L), Freundlich (F), Temkin (T), Dubinin-Radushkevich (D-R), and Sips (S). Finally, the kinetic equations used are pseudo-first order (PFO), pseudo-second order (PSO), intra-particle diffusion (I-PD), and Elovich (ELV).

#### 3.2.1. Uranium Compounds—PH Effect

**Figure 7:**
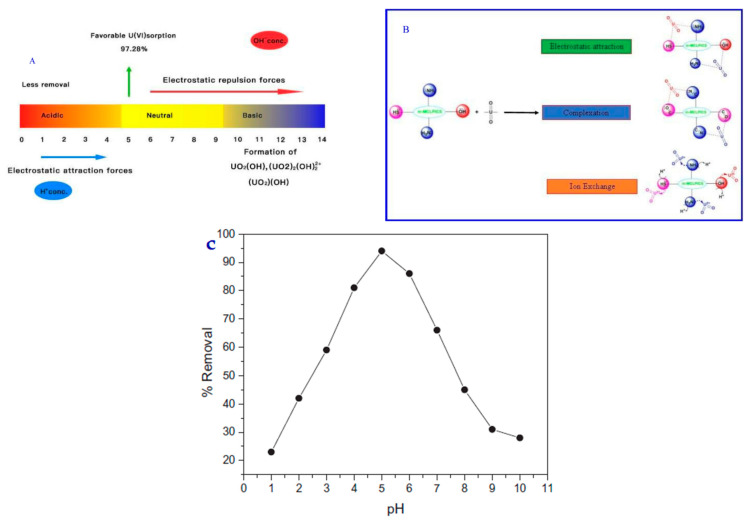
(**A**,**B**) pH mechanism with U(VI) ions; (**C**) pH effect on adsorption efficiency (%) of U(VI) ions from synthesized aqueous solution onto m-MCLPICS composite nanoadsorbent. Reprinted from Ref [11]. Copyright 2020 with permission from Elsevier B.V.

In the study of Gutha Yuvaraja et al., the pH effect (1.0–10.0) on the equilibrium pH solution, using an m-MCLPICS composite nanoadsorbent, is presented. The effect of the pH has an influence on the m-MCLPICS adsorbent, as well as the speciation of the adsorbate, and its mechanism is presented in Figure 7A–C. The experiments for the effect of the pH were achieved at an initial U(VI) concentration of 40 mg/L. More specifically, Figure 7C depicts the pH effect for values ranging from 1 to 10 for the uptake of U(VI) on the surface of the m-MCLPICS composite nanoadsorbent. In the case of lower values of pH, the removal percentage of U(VI) ions is low. In addition, when the pH values are below 4.0, the $OU_{2}^{2+}$ ions are the dominant species in the synthesized aqueous solution system. In addition, in the case of pH values lower than 4.0, most of the active sites of the composite nanoadsorbent are protonated, which results in the relatively low uptake amounts of U(VI) ions from the synthesized aqueous solution. The concentration of H^+^ ions, in the case of low pH levels (acidic conditions), is very high, and this may result in the formation of an ion exchange mechanism. However, from the experiments of the pH effect, it can be clearly observed that the removal percentage of U(VI) ions on the surface of m-MCLPICS increases for pH values from 2 to 5 (optimum pH 5). The selected functional groups of the composite nanoadsorbent and their involvement in the system of the U(VI) adsorption mechanism using m-MCLPICS was elucidated well, with electrostatic attraction and complexation/coordination due to ion exchange. In addition, it must be noted that the oxygen and nitrogen compounds have a lone pair of electrons, and, consequently, can form a complex with the U(VI) ions via a pair of electrons sharing. Thus, the surface complexation that occurs during the process of U(VI) adsorption is attributed to the aforementioned reasons (electrons). In addition, another reason for the surface complexation is electrostatic attraction due to the positive charge of $OU_{2}^{2+}$ and negative charge of the m-MCLPICS composite nanoadsorbent. During the adsorption mechanism, the groups of carboxyl were deprotonated and the surface of the m-MCLPICS composite nanoadsorbent obtained a charge with a negative sign, which resulted in the attraction of U(VI) ions via electrostatic attraction forces. However, the removal percentage during the attraction of U(VI) ions via electrostatic forces was less. Moreover, it was observed that, for pH > 5, the removal percentage of U(VI) ions on the surface of m-MCLPICS composite nanoadsorbent decreases. Thus, at a pH > 5, electrostatic repulsion occurs, due to deprotonation between the negatively charged hydroxyl uranyl and negatively charged surface of the m-MCLPICS composite nanoadsorbent. Thus, all of the experimental studies of U(VI) adsorption were achieved at pH 5.0 [11].

**Figure 8:**
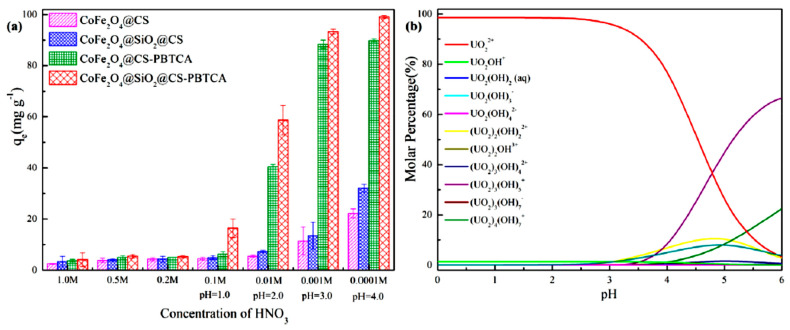
(**a**) Acidity effect on the U(VI) adsorption onto (CoFe_2_O_4_@CS, CoFe_2_O_4_@SiO_2_@CS, CoFe_2_O_4_@CS-PBTCA) CoFe_2_O_4_@SiO_2_@CS-PBTCA; (**b**) U(VI) model pollutant distribution in aqueous solution with C_0_ = 100 mg/L and standard pH values (0–6) calculated using Visual MINTEQ 3.1. Reprinted from Ref [9]. Copyright 2020 with permission from Elsevier B.V.

In the study of Yaoyao Huang et al., the effect of solution acidity on U(VI) adsorption using a (CoFe_2_O_4_@CS, CoFe_2_O_4_@SiO_2_@CS, CoFe_2_O_4_@CS-PBTCA) CoFe_2_O_4_@SiO_2_@CS-PBTCA composite nanoadsorbent was investigated. The effect of solution acidity on the efficiency of the adsorption process was investigated with a pure U(VI) solution (100 mg/g) at a wide variety of pH values (0-6). In addition, considering that the U(VI) ions precipitate in the case of high pH values, and also because the researchers wanted to keep the stability of the CoFe_2_O_4_@SiO_2_@CS-PBTCA composite nanoadsorbent, the experiments of adsorption were carried out at solution acidities ranging from 0.0001 M to 1.0 M at pH 4.0. Figure 8a below presents that, in the case where the solution acidity was adjusted from 0.0001 M to 1.0 M (pH 4.0), the efficiency of the adsorption capacity decreased because the functional groups of the CoFe_2_O_4_@SiO_2_@CS-PBTCA composite nanoadsorbent presented decreased protonation, resulting in a gradual decrease in the coordination ability of U(VI) ions. However, the higher adsorption capacity was achieved using the CoFe_2_O_4_@SiO_2_@CS-PBTCA composite nanoadsorbent at a pH of 4.0 (optimum pH), and a total concentration of U(VI) ions of 100 mg/g. The pH values were calculated using Visual MINTEQ 3.1. In addition, the acidity of the synthesized solution affects not only the efficiency of the adsorption of the synthesized composite adsorbent, but also the magnetic core stability. The magnetic nanoparticles of chitosan have a low stability in the presence of environments with high acidity, where the CoFe_2_O_4_ magnetic core is easily destroyed and cobalt and iron ions are leached, resulting in secondary pollution and a loss of magnetic properties (pH < 4.0). However, it was found that almost no cobalt and iron leaching was discovered over the entire range of acidity (pH 4.0 to 1.0 M) from CoFe_2_O_4_@SiO_2_@CS-PBTCA, provided that the synthesized composite nanoadsorbent possessed a good resistance in acidic media after coating with silica [9]. 

#### 3.2.2. Uranium Compounds—Evaluation of Adsorption Isotherm Models

The best mathematical fitting of experimental data for the uptake of U(VI) ions from the aqueous solution onto m-MCLPICS was achieved using the L model (R^2^ = 0.999). This result indicates that the m-MCLPICS functional groups were uniformly occupied by the metal ions of the U(VI) model pollutant and the main mechanism was the homogeneous or monolayer adsorption. However, the highest adsorption capacity (Q_max_) of U(VI) ions onto m-MCLPICS was found to be 250.7 mg/g at 303 K. It must be noted that the F model presents lower R^2^ and K_F_ values, indicating that the adsorption process is not heterogeneous and also that the F model is not suitable for the adsorption of U(VI) ions. In addition, the R_L_ factor using m-MCLPICS was found to be 0.125, which is a result that suggests a favorable adsorption of U(VI) ions. Furthermore, the isotherm model of D-R was also fitted to the experimental data in order to obtain results about the nature of adsorption processes. In addition, the mean free energy for the adsorption of U(VI) ions can be determined according to the following equation:

(3) $$E\;=\frac{1}{\sqrt{2K}}$$

It must be noted that the mechanism of adsorption can be clearly described with the values of free energy. Moreover, in the case where the obtained values of E existed between 1 and 8 kJ/mol, the process of adsorption can be characterized as physisorption processes, and when the value of E lies between 8–16 kJ/mol, the process of adsorption can be considered as ion exchange. In the present study, after the calculation, it was found that the free energy values are between 2.012 and 3.012 kJ/mol, respectively, indicating a physisorption process. In the below Table 4, the different parameters (R^2^ and χ^2^) obtained from the used isotherm models are presented [11].

**Figure 9:**
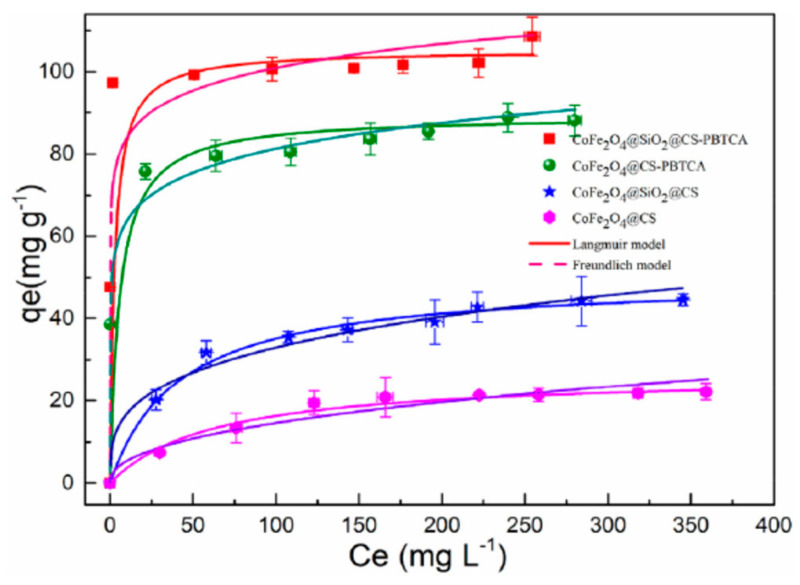
U(VI) adsorption isotherms and corresponding L and F models. Reprinted from Ref [9]. Copyright 2020 with permission from Elsevier B.V.

The effect of different initial U(VI) concentrations on the efficiency of adsorption process was also examined from 50 to 400 mg/L, in order to determine the saturated capacity of adsorption of the CoFe_2_O_4_@SiO_2_@CS-PBTCA composite nanoadsorbent at pH 4.0. The amount of U(VI) ions that adsorbed per unit mass using CoFe_2_O_4_@SiO_2_@CS-PBTCA, from 47.68 to 104.56 mg/g, was the largest when compared with the other synthesized magnetic chitosan composite nanoadsorbent (Figure 9). In the case where the initial concentration increased from 50 to 100 mg/L, the adsorption capacity at equilibrium increased significantly. This result revealed that the low initial U(VI) concentration was the key driving force in reducing the mass transfer resistance of U(VI) ions between the solid and liquid phases. However, when the initial U(VI) concentration was higher than 150 mg/L, no significant increase in adsorption capacity was found. This result may be attributed to the fact that, at higher concentrations of U(VI) ions, the active sites on the CoFe_2_O_4_@SiO_2_@CS-PBTCA composite nanoadsorbent tend to be saturated and cannot bind the model pollutant [9].

#### 3.2.3. Uranium Compounds—Adsorption Kinetics

**Figure 10:**
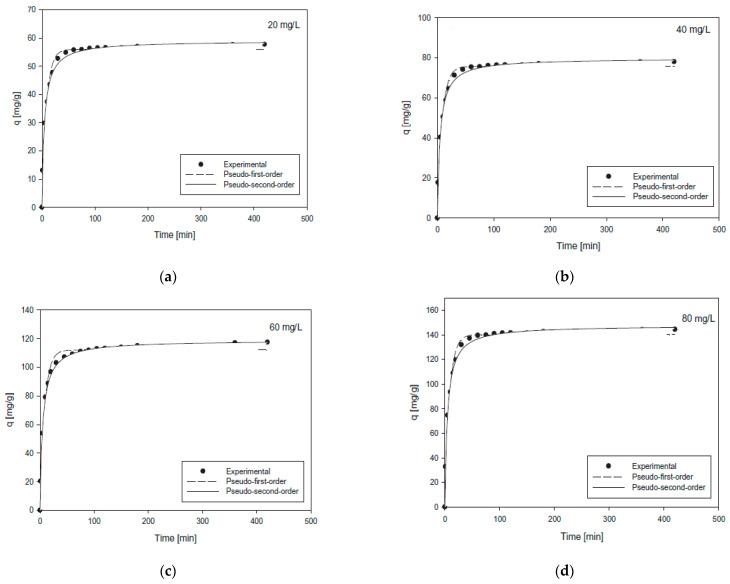
(**a**–**d**) Evaluation of kinetic plots (20, 40, 60, and 80 mg/L) (contact time: 90 min, U(VI) concentration: 20–80 mg/L, pH 5.0, amount of dose: 0.5 g, temperature: 303 K, amount of solution: 50 mL). Reprinted from Ref [11]. Copyright 2020 with permission from Elsevier B.V.

The kinetic parameters k_1_, k_2_, and R^2^, which were obtained using the non-linear regression of PFO and PSO kinetic models, are presented in Table 5. Furthermore, in Figure 10a–d the kinetic curves that were obtained from the non-linear adsorption, in the case of the U(VI) ions uptake from aqueous solutions, are presented. The relatively high values of correlation coefficients in the case of PSO (R^2^ = 0.9949–0.9983) indicate that the dominant mechanism in this experimental process is chemisorption. However, the relatively low values of the PFO (R^2^ = 0.9714–0.9874) kinetic model, when compared with the values of PFO, indicate that this model is not appropriate for adsorptive analysis. Hence, the rate-controlling step (chemisorption) might be involved in the forces of valence via the sharing or exchange of electrons between U(VI) ions and the m-MCLPICS composite nanoadsorbent [11].

**Figure 11:**
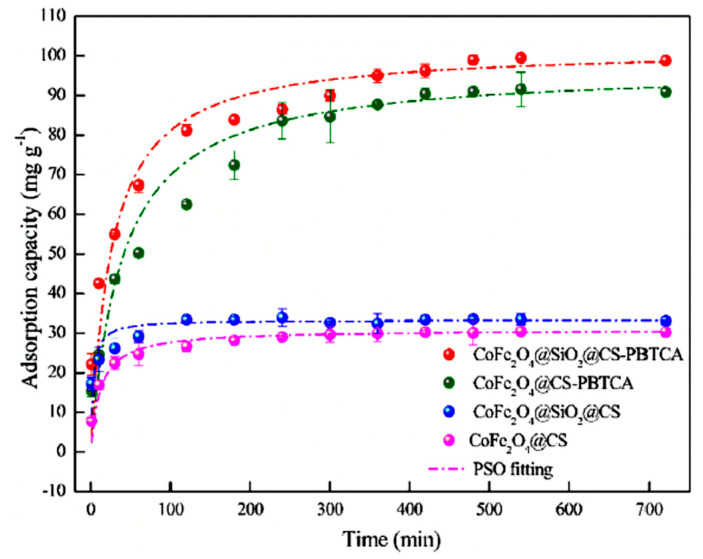
Contact time effect and fitting of PSO model for the adsorption of U(VI) ions onto CoFe_2_O_4_@SiO_2_@CS-PBTCA. Reprinted from Ref [9]. Copyright 2020 with permission from Elsevier B.V.

The kinetics of adsorption were tested by studying the adsorption of U(VI) ions, at pH 4.0, at different times. In Figure 11 (red and green lines), it can be clearly observed that the effectiveness of adsorption capacity after grafting with 2-phosphonobutane-1,2,4-tricarboxylic acid was much higher when compared with the nongrafted nanoparticles of magnetic chitosan (blue and pink lines). The efficiency of the adsorption capacity for silica-uncoated adsorbents was lower when compared with those of silica-coated adsorbents. Meanwhile, the adsorption equilibrium for the CoFe_2_O_4_@SiO_2_@CS-PBTCA composite nanoadsorbent was reached after 8 h and 80% of the total adsorption of U(VI) ions was achieved at 6 h. After the fitting of experimental data to the PFO, PSO, and I-PD models, it was found that the best mathematical fitting, in the case of the CoFe_2_O_4_@SiO_2_@CS-PBTCA composite nanoadsorbent, was achieved with the PSO model (R^2^ = 0.9974), indicating a chemisorption process. Meanwhile, the divergence between q_e,cal._ (102.04 mg/g) and q_e,exp._ (99.47 mg/g) was very low for the CoFe_2_O_4_@SiO_2_@CS-PBTCA composite nanoadsorbent. Finally, the general conclusion from this research study was that the high adsorption capacity of CoFe_2_O_4_@SiO_2_@CS-PBTCA is attributed to the chemical interaction of the U(VI) model pollutant with the surface phosphonic groups and carboxyl groups of the composite adsorbent [9].

### 3.3. Hg (II) Removal

Below, several selected studies for the uptake of mercury (Table 6) from synthetic aqueous solutions by using composite adsorbents derived from chitosan are presented. In addition, the pH (optimum), preferred, and best mathematical fitting of isotherms models and kinetics equations, and also the adsorption capacity (Qmax) of the selected composite adsorbents derived from chitosan are presented. The isotherm models used are L, F, T, D-R, university of Tehran isotherm (UT), Redlich—Peterson (R-P), and Radke-Prausnitz (R-P*). Finally, the kinetic equations used are PFO, PSO, I-PD, and ELV.

#### 3.3.1. Mercury Compounds—PH Effect

The process of metal ions removal from an aqueous solution is based on the value of the solution’s pH. The efficiency of the process is attributed to the charges of the functional groups of composite adsorbents, due to both the varying of the pH value (acidic-basic) and the form of the transition metal ions species in the aqueous solution. In the study of Nacer Ferrah, the pH effect (1.0–4.7) on the equilibrium pH solution, using an MCFs-EDTA-Na_2_ composite adsorbent, is presented. In addition, the maximum uptake of Hg(II) ion using the MCFs-EDTA-Na_2_ was achieved at an equilibrium pH of approximately 4.69. In addition, in the case of pH < 3, the binding sites of the surface of the composite adsorbent obtains a positive charge due to the protonation reaction, resulting in a decrease in the sorption process because of the repulsion effect. However, the excess of protons in the aqueous solution leads to high competition between H_3_O^+^ and mercury(II) ions onto the available binding sites of the composite adsorbent [22].

**Figure 12:**
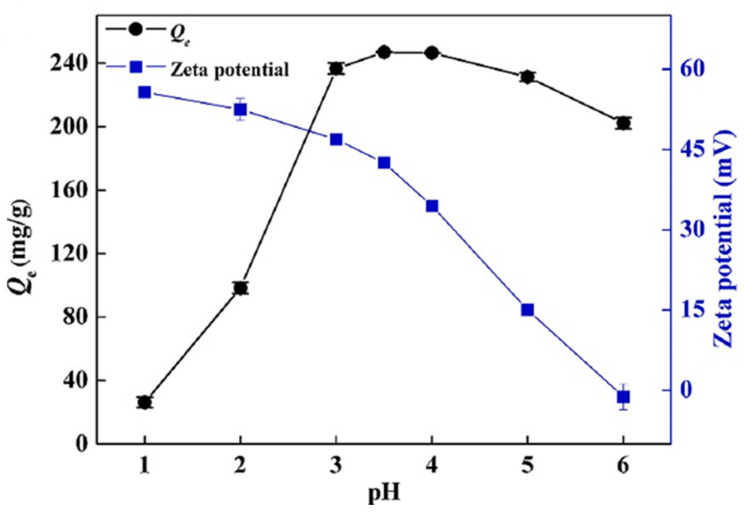
pH effect on adsorption of Hg(II) metal ions and zeta potentials onto MCTP composite adsorbent. Reprinted from Ref [25]. Copyright 2020 with permission from Elsevier B.V.

In another study by Yong Fu et al., in Figure 12, the pH effect on the adsorption of Hg (II) metal ions, and also the trend of zeta potentials on the synthesized MCTP polymer adsorbent, is presented. During the experimental process, it was observed that, at pH values between 2 and 3.5 (3.5 optimum pH), the highest adsorption capacity of Hg(II) metal ions was revealed. Despise the aforementioned, at a pH value higher than 3.5, the adsorption capacity decreased. In the case of the zeta potential graphical abstract, the obtained values (mV) gradually decreased when the value of the pH ranged between 1 and 6, indicating that the active surface sites of the synthesized polymer composite adsorbent (MCTP) obtains a positive charge of its functional groups at pH 5.9. In addition, according to the obtained results, the pH effect on the adsorption of Hg(II) metal ions in the case of low pH values (1–2) is attributed to the high concentration of H^+^ in aqueous solution, which, in turn, generates the occupation of binding sites of the MCTP polymer composite adsorbent, resulting in the creation of electrostatic repulsion between the Hg(II) metal ions and the surface functional groups of MCTP, hindering the coordinated complex formation of Hg(II) metal ions.

The rapid increase in adsorption capacity in the case of pH values 1–3.5, and the gradual decrease in zeta potential values, is attributed to the decrease in the degree of protonation and electrostatic repulsion of the functional groups. Moreover, at higher pH values (4–6), the phenomenon of a formation of Hg(II) hydroxide species, including Hg(OH)^+^ (soluble) and Hg(OH)_2_ (insoluble precipitate), occurs, resulting in the blockage of the active site of pores and of MCTP, and thus the consequent decrease in the overall adsorption capacity. However, the optimum adsorption capacity (mg/g) was achieved at pH 3.5 [25].

#### 3.3.2. Mercury Compounds—Evaluation of Adsorption Isotherms Models

When the value of R_L_ is in the range of 0–1, it is indicated that the L isotherm is favorable for the process of adsorption, whereas, in the case of Hg(II) removal using MCFs-EDTA-Na_2_, the composite adsorbent was in the range of 0.20–0.87. The R^2^ value that was obtained from the model of F was lower than L (R^2^ = 0.99), indicating a monolayer sorption of Hg(II) ion onto the surface of the MCFs-EDTA-Na_2_ composite adsorbent [22].

**Figure 13:**
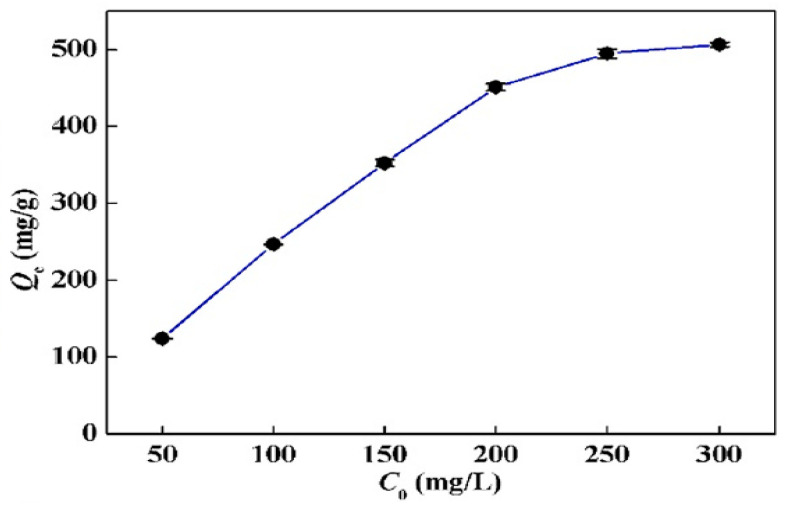
Effect of initial concentration on adsorption of Hg(II) ion onto MCTP composite adsorbent. Reprinted from Ref [25]. Copyright 2020 with permission from Elsevier B.V.

In Figure 13, it is presented that the adsorption capacity increased quickly in the case of 50–200 mg/L Hg(II) ion concentration, whereas, in the case of a concentration >250 mg/L, the adsorption capacity of the Hg(II) ion increased slowly, and the value of the equilibrium maximum removal obtained was 506.39 mg/g. This slow increase in adsorption capacity (250–300 mg/g) is attributed to the complete occupation of MCTP surface active groups. According to the obtained correlation coefficients (R^2^), it was found that the F isotherm model (R^2^ = 0.9159) does not have as good a mathematical fitting as the isotherm model of L (R^2^ = 0.9988). However, the obtained value of Q_max, exp_ (515.46 mg/g) is in agreement with the L isotherm model (Q_max,cal_ = 506.39 mg/g), while the good mathematical fitting of the L isotherm model indicates a monolayer adsorption of Hg(II) metal ions on the surface of MCTP. Thus, according to the experimental results, it can be concluded that, with the use of MCTP in acidic aqueous media, the Hg(II) metal ions from an aqueous solution can be removed [25].

#### 3.3.3. Mercury Compounds—Adsorption Kinetics

The data obtained from the kinetic study are used in order to explain the solute sorption rate. The sorption of metal species is not an instantaneous process because the solid-liquid extractions involve several steps: (i) metal species diffusion from the bulk solution to the surface of the adsorbent, (ii) metal species diffusion in the internal surface areas, and (iii) the generated chemical reaction between the surface functional groups of the adsorbent and metal species. The kinetics of the Hg(II) ion on the surface of MCFs-EDTA-Na_2_ was investigated using PFO, PSO, ELV, and I-PD models. However, according to the experimental results, it was revealed that the best mathematical fitting was obtained using the PSO (Figure 2) [22].

From the values of R^2^, it was found that the PSO model was more adequate when compared with the other theoretical models of ELV, PFO, and I-PD. Consequently, the order of models according to the value of R^2^ was PSO > ELV > PFO > I-PD. However, a high deviation was found between the values of Q_exp._ and Q_cal._ using the models of ELV and I-PD, respectively [22].

The kinetics of the Hg(II) ion on the surface of MCTP was investigated using PFO, PSO, and I-PD models. According to the experimental results, it was revealed that the best mathematical fitting was obtained using the PSO (R^2^ = 0.9998), whereas, in the case of PFO, the value was lower (R^2^ = 0.7305), indicating the chemical reaction between the adsorbate and adsorbent, due to the sharing or exchange of electrons.

**Figure 14:**
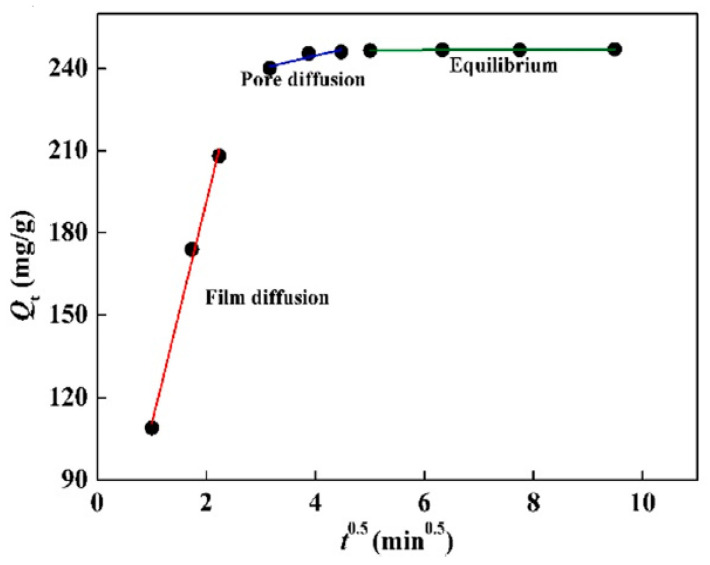
Fit of kinetic data derived from the experimental process to I-PD model. Reprinted from Ref [25]. Copyright 2020 with permission from Elsevier B.V.

In addition, the I-PD model was used for the determination and analysis of experimental data. Figure 14 presents the obtained results after the fitting of experimental data. However, it can be clearly observed that the process of Hg(II) removal can be separated into three phases, including film diffusion, pore diffusion, and equilibrium. The first straight line indicates the film diffusion and the quick adsorption of Hg(II) from the synthesized aqueous solution on the surface of MCTP, while the I-PD process occurs when the Hg(II) ion is transported slowly to the interior structure of MCTP through a pore diffusion process. The final stage of equilibrium can be achieved with the complete occupation of the available binding sites [25].

### 3.4. Rare Earth Elements

Below, several selected studies for the uptake of REEs (Table 7) from synthetic aqueous solutions by using composite adsorbents derived from chitosan are presented. In addition, the pH (optimum), preferred, and best mathematical fitting of isotherms models and kinetics equations, and also the adsorption capacity (Q_max_) of the selected composite adsorbents derived from chitosan, are presented. The isotherm models used are L, F, D-R, and S. Finally, the kinetic equations used are PFO, PSO, I-PD, and ELV.

#### 3.4.1. Rare Earth Elements—PH Effect

**Figure 15:**
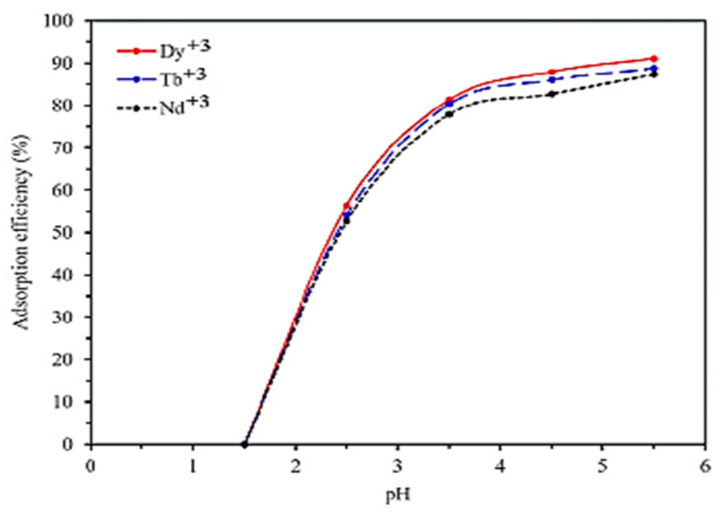
pH effect on adsorption efficiency (%) of Dy(III), Tb(III), and Nd(III) rare earth metal ions from synthesized aqueous solution onto CA/CMC/Ni_0.2_Zn_0.2_Fe_2.6_O_4_ composite adsorbent. Reprinted from Ref [35]. Copyright 2020 with permission from Elsevier B.V.

In the study of Hamedreza Javadiana et al., the solution’s pH is referred to have a key role in the process of adsorption because it can affect the adsorption capacity, solution chemistry, metal speciation, activity of functional groups of adsorbent, and the generated mechanism of adsorption. In Figure 15, it is presented that the values of the solution’s pH affect the adsorption efficiency of the selected model pollutants Dy(III), Tb(III), and Nd(III) on the surface of the CA/CMC/Ni_0.2_Zn_0.2_Fe_2.6_O_4_ composite nanoadsorbent. The electrostatic interactions between the surface of the CA/CMC/Ni_0.2_Zn_0.2_Fe_2.6_O_4_ composite adsorbent and the forming of species of rare earth elements are tremendously affected by the pH value and continuously increase for pH values 1.5–5.5 (optimum pH 5.5). For a pH value of 1.5, a low removal of Dy(III), Tb(III), and Nd(III) metal ions is presented due to the protonation of the functional groups of the CA/CMC/Ni_0.2_Zn_0.2_Fe_2.6_O_4_ composite adsorbent, resulting in the blocking of binding sites (zero adsorption efficiency) due to the presence of H^+^. However, for pH values 2.5–5.5, the adsorption efficiency for Dy(III), Tb(III), and Nd(III) on the surface of CA/CMC/Ni_0.2_Zn_0.2_Fe_2.6_O_4_ increases due to the reduction in competition between model pollutant ions and H^+^ ions. In addition, the adsorption efficiency of the composite adsorbent was not examined at a pH value higher than 5.5 due to the precipitation of the model pollutant ions in the form of hydroxide [35].

**Figure 16:**
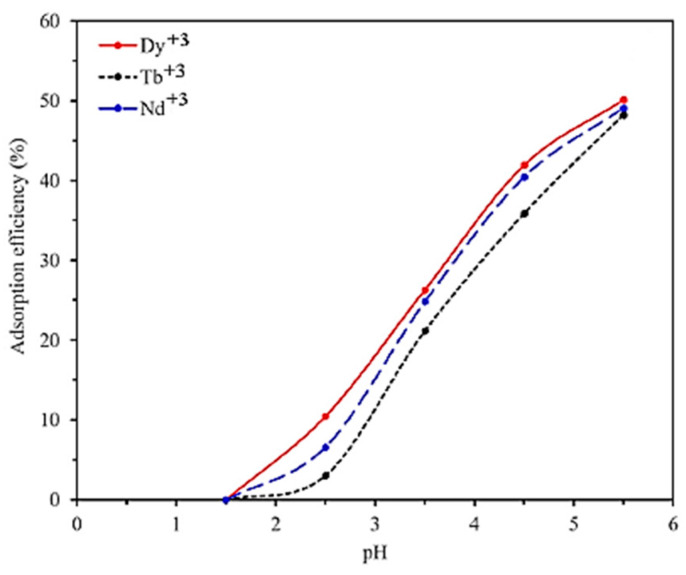
pH effect on adsorption efficiency (%) of Dy(III), Tb(III), and Nd(III) rare earth metal ions from synthesized aqueous solution onto CMC/P(PTA)/Ni_0.2_Zn_0.2_Fe_2.6_O_4_ composite nanoadsorbent. Reprinted from Ref [37]. Copyright 2020 with permission from Elsevier B.V.

In another study by the same author, as aforementioned above, the removal of Dy(III), Tb(III), and Nd(III) rare earth metal ions from a synthesized aqueous solution using a CMC/P(PTA)/Ni_0.2_Zn_0.2_Fe_2.6_O_4_ composite nanoadsorbent is examined. In Figure 16, it is presented that the values of the solution’s pH affect the adsorption efficiency of the selected model pollutants Dy(III), Tb(III), and Nd(III) on the surface of the CMC/P(PTA)/Ni_0.2_Zn_0.2_Fe_2.6_O_4_ composite adsorbent. The electrostatic interactions between the surface of the CMC/P(PTA)/Ni_0.2_Zn_0.2_Fe_2.6_O_4_ composite adsorbent and the forming of species of rare earth elements are tremendously affected by the pH value and continuously increase for pH values 1.5–5.5 (optimum pH 5.5), which is a result that is attributed to the influence of pH to the solubility of selected metal ions in the aqueous solution and the ionization status of the groups on the surface of the CMC/P(PTA)/Ni_0.2_Zn_0.2_Fe_2.6_O_4_ composite nanoadsorbent. In addition, the obtained experimental data suggests that the adsorption efficiency of metal ions on the surface of the composite nanoadsorbent increase for a pH value higher than 1.5 because the interaction between metal cations and hydrogen for capturing the active sites is reduced. The low degree of protonation of functional groups of the composite nanoadsorbent also inhibits the relation between model pollutants and the adsorbent, which could be the main reason for the low adsorption efficiency in the case of low pH values [37].

#### 3.4.2. Rare Earth Elements—Evaluation of Adsorption Isotherms Models

In Table 8, the obtained values of R^2^ using L and F isotherm models, the value of RL, etc., are presented. In the case of the adsorption of Tb(III) and Dy(III) metal ions from the aqueous solution using the CA/CMC/Ni_0.2_Zn_0.2_Fe_2.6_O_4_ composite adsorbent, it was found that the model of F has a better mathematical fitting when compared with the L model, with R^2^ values of 0.9905 and 0.9751, respectively, which is a result that indicates the adsorption of the aforementioned model pollutant ions on heterogeneous sites. Now, in the case of the Nd(III) model pollutant ions, the best mathematical fitting was achieved with the model of L (R^2^ = 0.9703), indicating that the surface adsorption mechanism of the L model controls the Nd(III) adsorption on the surface of the CA/CMC/Ni_0.2_Zn_0.2_Fe_2.6_O_4_ composite adsorbent. More specifically, this result indicates that the process of adsorption is limited only to the adsorption sites of the composite adsorbent, forming a monolayer of Nd(III) ions on the surface of CA/CMC/Ni_0.2_Zn_0.2_Fe_2.6_O_4_, and when the covering of active sites from the model pollutant is achieved, no further adsorption occurs. From Table 8, it can be observed that the values of the R_L_ factor for the removal of Dy(III), Tb(III), and Nd(III) ions from the aqueous solution on the CA/CMC/Ni_0.2_Zn_0.2_Fe_2.6_O_4_ composite adsorbent indicates a favorable adsorption process because is between 0 and 1. However, the increasing trend of n values (higher than 1) for all cases of model pollutant removal represents a favorable adsorption condition [35].

According to the adsorption constants of the L model (Table 9), it was found that the R_L_ values for Dy(III), Tb(III), and Nd(III) ions were 0.0005, 0.0022, and 0.001, respectively, indicating that the process of adsorption is favorable because these values are between 0 and 1. Moreover, according to the model of F, it can be clearly observed that the 1/n values for Dy(III), Tb(III), and Nd(III) ions were 0.12, 0.13, and 0.09, respectively, representing highly curved isotherms because the 1/n values are lower than 0.7. The results from the experimental data for the adsorption of Dy(III), Tb(III), and Nd(III) ions on CMC/P(PTA)/Ni_0.2_Zn_0.2_Fe_2.6_O_4_ are well fitted to the model of the F isotherm, with R^2^ = 0.9632, 0.9482, and 0.9709, respectively. In addition, the obtained results for each model pollutant are confirmed due to the lower values of χ^2^. Therefore, as general result, according to the fitting experimental data, it can be concluded that the process of adsorption of the metal ions on the surface of CMC/P(PTA)/Ni_0.2_Zn_0.2_Fe_2.6_O_4_ is performed by the adsorbent sites that are heterogeneous, indicating multilayer adsorption (non-uniform) [37].

#### 3.4.3. Rare Earth Elements—Adsorption Kinetics

Table 10 presents the kinetic constants for the adsorption of Dy(III), Tb(III), and Nd(III) ions on the CA/CMC/Ni_0.2_Zn_0.2_Fe_2.6_O_4_ composite adsorbent, using PFO, PSO, and I-PD kinetic models. The theoretical values of q_e_ (q_e,cal._) derived from the PFO model in Dy(III), Tb(III), and Nd(III) adsorption are not in agreement with the experimental values of q_e_ (q_e,exp._), whereas, when using the PSO kinetic model, the q_e,cal._ values are close to q_e,exp._ However, according to R^2^, the PSO model has a better mathematical fitting to the experimental data than the PFO, for all cases of model pollutants. Therefore, the adsorption process of Dy(III), Tb(III), and Nd(III) ions on the CA/CMC/Ni_0.2_Zn_0.2_Fe_2.6_O_4_ composite adsorbent are well described by the PSO model, indicating that the rate-limiting step in selected metal ions model pollutants is the chemisorption process, which involves valence forces by sharing or exchanging electrons between the metal ions and the surface of the composite nanoadsorbent [35].

The kinetics of Dy(III), Tb(III), and Nd(III) ions on the surface of CMC/P(PTA)/Ni_0.2_Zn_0.2_Fe_2.6_O_4_ was investigated using PFO, PSO, and I-PD models. In addition, it was found that the R^2^ values obtained from the PSO for Dy(III), Tb(III), and Nd(III) ions were 0.9686, 0.9564, and 0.9629, which are greater when compared with those of the PFO model (0.8803, 0.8538, and 0.8831). Furthermore, the χ^2^ values of the PSO model are also lower than those of the PFO model. In addition, the values of q_e,exp_ that were obtained from the PSO model are in agreement with the values of q_e,cal_, indicating a chemisorption process. Furthermore, the values of χ^2^ and R^2^ that were obtained after the fitting of the I-PD model to the empirical data indicate that, except from the PSO model, the adsorption kinetics of Dy(III), Tb(III), and Nd(III) ions on the surface of CMC/P(PTA)/Ni_0.2_Zn_0.2_Fe_2.6_O_4_ are also well described by the I-PD model. Thus, the adsorption processes of Dy(III), Tb(III), and Nd(III) ions on the composite nanoadsorbent follow both PSO and I-PD kinetic models (Table 11) [37].

### 3.5. Adsorption Mechanisms for the Removal of Uranium from Aqueous Solution

**Figure 17:**
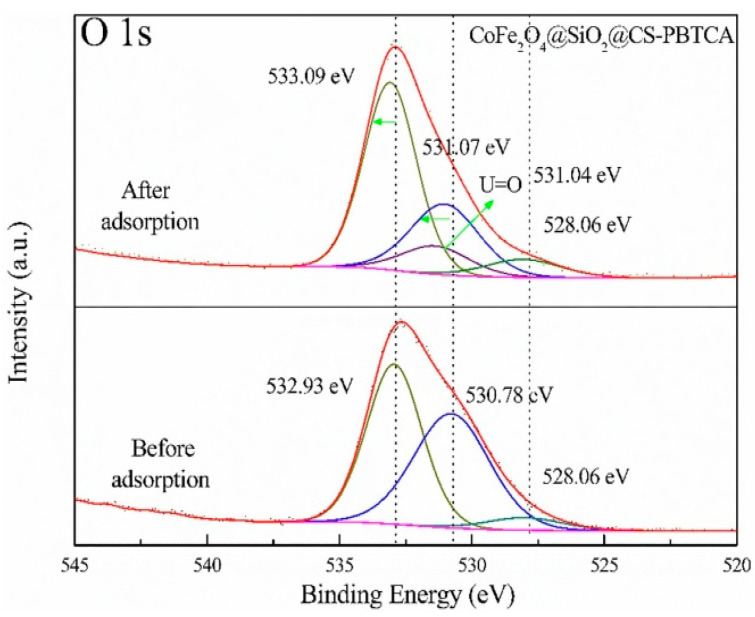
The high resolution O 1s XPS spectra of CoFe_2_O_4_@SiO_2_@CS-PBTCA composite nanoadsorbent after and before U(VI) adsorption. Reprinted from Ref [9]. Copyright 2020 with permission from Elsevier B.V.

Below, some selected studies for the adsorption of U(VI) onto chitosan composite nanoadsorbents are presented. In the study of Yaoyao Huang et al., the possible adsorption mechanism of U(VI) onto the CoFe_2_O_4_@SiO_2_@CS-PBTCA composite nanoadsorbent was investigated. The organology used was XPS and FTIR analysis. To examine the interactions between the CoFe_2_O_4_@SiO_2_@CS-PBTCA functionalized groups and the U(VI) model pollutant, a deconvolution analysis of the O 1s and N 1s spectra was conducted. The spectrum of O 1s (Figure 17) of the CoFe_2_O_4_@SiO_2_@CS-PBTCA composite nanoadsorbent before the adsorption of U(VI) can be deconvoluted into three individual peaks, with binding energies of 532.93, 530.78, and 528.06 eV, which were attributed to C=O/C-O, P=O/P-O, and to O atoms in Fe-O, respectively. In addition, after the adsorption of U(VI) onto the CoFe_2_O_4_@SiO_2_@CS-PBTCA composite nanoadsorbent, the peaks of O 1s of the C=O/C-O and P=O/P-O groups shifted to higher energies of binding, with values of 533.09 and 531.07 eV, respectively. Moreover, a new peak can be clearly observed at 531.04 eV, which corresponded to U=O. These results demonstrate that O atoms in P=O/P-O and C=O/C-O groups underwent complexation with the metal ions of U(VI) by sharing electrons in order tο form U=O bonds.

**Figure 18:**
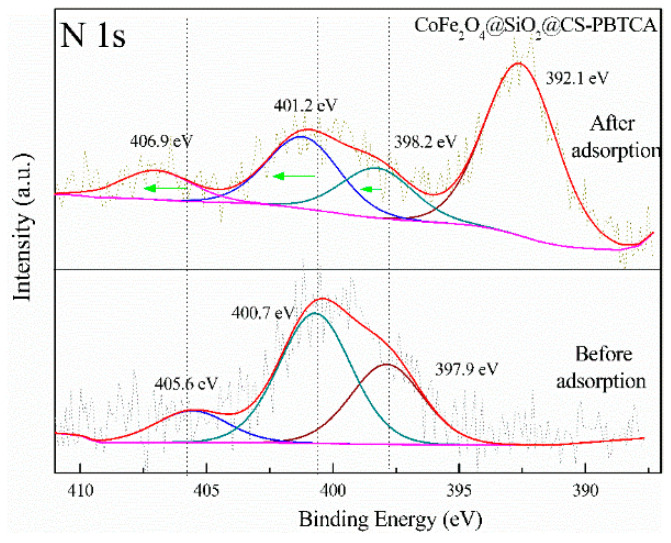
The high resolution N 1s XPS spectra of CoFe_2_O_4_@SiO_2_@CS-PBTCA composite nanoadsorbent after and before U(VI) adsorption. Reprinted from Ref [9]. Copyright 2020 with permission from Elsevier B.V.

Furthermore, before the adsorption of U(VI) into the CoFe_2_O_4_@SiO_2_@CS-PBTCA composite nanoadsorbent, the N 1s spectrum (Figure 18) was resolved into three component signals attributed to NH_3_^+^ (405.6 eV), NH/NH_2_ (400.7 eV) and N-C (397.9 eV) species, whereas, after the adsorption of U(VI), the N 1s peaks of the NH_3_^+^, NH/NH_2_, and N-C groups were slightly shifted to higher energies of binding. In addition, the obtained results confirmed that U(VI) also reacted with the atoms of N during the process of adsorption. In addition, it must be noted that the non-grafted adsorbent CoFe_2_O_4_@SiO_2_@CS (49.75 mg/g) had a lower adsorption capacity than the PBTCA-grafted adsorbent CoFe_2_O_4_@SiO_2_@CS-PBTCA (105.26 mg/g), which is a result that indicates that the phosphonic and carboxyl groups on the grafted PBTCA composite nanoadsorbent played significant roles in the high U(VI) adsorption capacity [9].

**Figure 19:**
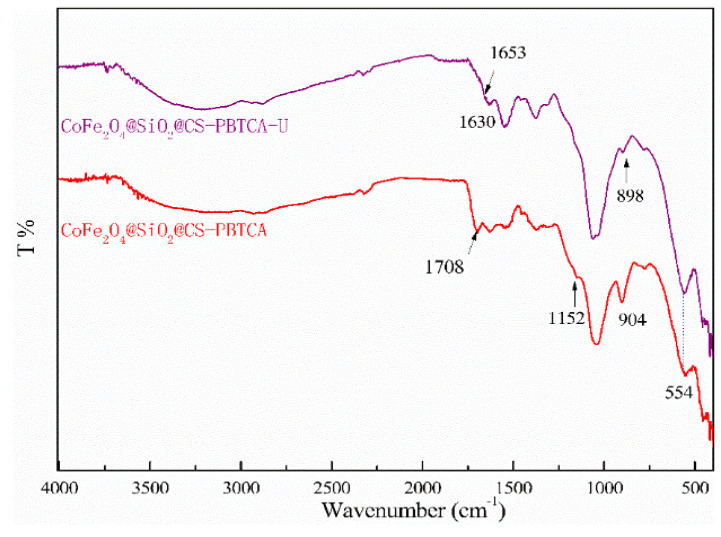
FTIR spectrum of the CoFe_2_O_4_@SiO_2_@CS-PBTCA before and after U(VI) uptake. Reprinted from Ref [9]. Copyright 2020 with permission from Elsevier B.V.

FTIR spectra was also used in order to clarify the surface functional groups onto the CoFe_2_O_4_@SiO_2_@CS-PBTCA composite nanoadsorbent before and after the adsorption of U(VI) (Figure 19). In addition, before the adsorption, the CoFe_2_O_4_@SiO_2_@CS-PBTCA showed a peak at 1708 cm^−1^ (C=O), which, after loading with U(VI), shifted to 1653 cm^−1^, which is a result that indicates that the carboxyl groups (C=O) interact with the U(VI) model pollutant. Besides, the characteristic peak of P=O at 1152 cm^−1^ disappeared, whereas the peak of P-O at 904 cm^−1^, after U(VI) removal, was shifted to 898 cm^−1^. The aforementioned results demonstrate that the phosphonic and carboxyl groups played a major role in the adsorption of U(VI). In addition, analysis via FTIR further demonstrated the successful capture of U(VI) by the phosphonic and carboxyl groups in PBTCA, which are results that are in good agreement with the XPS measurements (see above). These results suggested that the atoms of O of phosphonic groups (P=O/P-O) and carboxyl groups (C=O/C-O) in PBTCA played essential roles in obtaining a high U(VI) capture, whereas the N atoms onto the surface of chitosan might also play a role in the uptake of U(VI). Determining the synergistic effect of amino, phosphonic, and carboxyl groups bound to the CoFe_2_O_4_@SiO_2_@CS-PBTCA composite nanoadsorbent is the key factor to ensuring the excellent properties of chelating for the removal of U(VI) [9].

**Figure 20:**
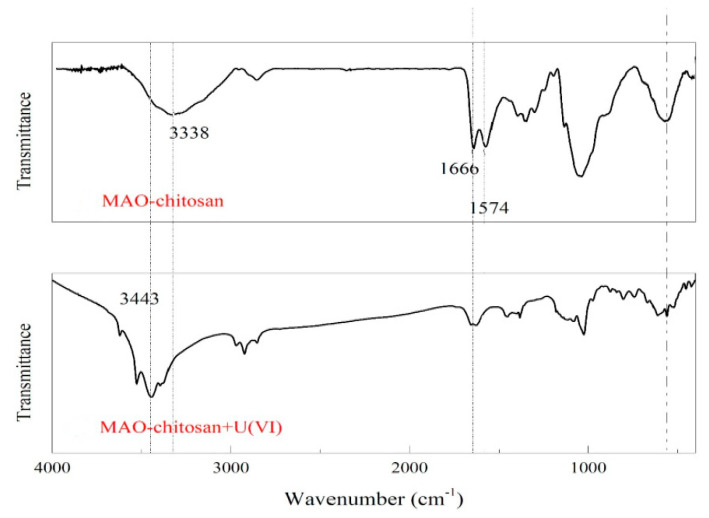
The FTIR spectra of MAO–chitosan. Reprinted from Ref [12]. Copyright 2018 with permission from Elsevier Ltd.

In another study by Zhuang et al., the possible adsorption mechanism of U(VI) onto the MAO-chitosan composite nanoadsorbent was investigated. In Figure 20, the FTIR spectra of the MAO-chitosan composite nanoadsorbent before and after uptake of U(VI) is presented. Therefore, in the case of the MAO-chitosan composite adsorbent after uptake of U(VI), it showed weaker peaks of 1666 cm^−1^ and 1574 cm^−1^ corresponding to C=N vibrating and amino vibrating, respectively, when compared with the raw MAO-chitosan. Moreover, the FTIR absorption peak of 3338 cm^−1^, which corresponds to -OH vibration, was red-shifted into 3443 cm^−1^. The aforementioned results indicated that both N and O atoms of the groups of amidoxime may be involved in the process of adsorption [12].

**Figure 21:**
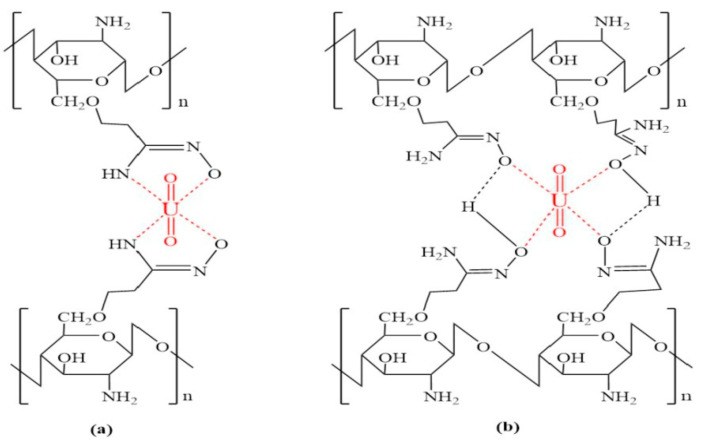
Possible mechanisms of adsorption of U(VI) onto MAO-chitosan composite nanoadsorbent. Reprinted from Ref [12]. Copyright 2018 with permission from Elsevier Ltd.

Therefore, according to the aforementioned analysis and other conducted research, in Figure 21, a mechanism of adsorption was tentatively proposed. At the given conditions, the UO_2_^2+^ was the main species that was derived from U(VI). If both the -OH and -NH_2_ of one group of amidoximes participated in the process of coordination, then two groups of amidoximes coordinating with one UO_2_^2+^, as depicted in Figure 21a, was likely. Whereas, if only -OH or -NH_2_ of one group of amidoxime was involved in the process of adsorption, then only one possible process of adsorption was likely, as presented in Figure 21b [12].

In addition, in another study by Xuejie Guo et al., the possible adsorption mechanism of U(VI) onto the FCCP composite nanoadsorbent was investigated. According to the studies of FTIR, the changes in the intensity of absorption peaks at approximately 1630, 1534, and 1159 cm^−1^ express the U(VI) coordination interplay with the groups of amide, C=O, and O-P-O. Moreover, a new vibrational band peak is revealed at 910 cm^−1^, indicating the existence of uranium. Subsequently, the FCCP composite nanoadsorbent elemental composition was further analyzed by XPS (before and after U(VI) adsorption). From the XPS spectra (full-range), after U(VI) removal, several new absorption peaks at characteristic energies of binding belong to U 4f_7/2_ (382.38 eV) and U 4f_5/2_ (393.28 eV) [20].

However, in order to verify the interaction between the FCCP composite nanoadsorbent and U(VI) model pollutant, the narrow scans for the peaks of O 1s and N 1s are analyzed in detail. The binding energies of O 1s and N 1s of FCCP-U are changed, when compared with those of the FCCP composite nanoadsorbent. Furthermore, it can be clearly observed that the adsorption of U(VI) from FCCP changes the energies of the binding of N 1s (~0.68 eV) between FCCP and FCCP-U. This result is possibly attributed to the chemical coordination of the U(VI) model pollutant with the chitosans’ N-atoms (N-H), leading to a distribution change in the surrounding environment of the electron. It must be noted that O 1s de-constructs into P-O, C=O, and -OH chemical states for FCCP-U and FCCP. More specifically, the energies of the binding of the -OH and P-O bonds shift to higher values (i.e., the -OH bond (~0.36 eV) and the P-O bond (~0.3 eV)). In contrast, the position of the C=O peak shifts only approximately 0.07 eV (<0.1 eV), which can be considered as immovability after the adsorption of U(VI), indicating that the bonds of coordination are composed mainly of nitrogen, phospholipid groups, and ions of U(VI). From the aforementioned, as a general result, it can be concluded that there are three types of interactions between the FCCP composite nanoadsorbent and U(VI), including electrostatic interaction, coordination, and intraparticle diffusion [20].

However, in another study by Mohammed F.Hamza et al., the possible adsorption mechanism of U(VI) onto the EPI-MG-CH composite nanoadsorbent was tested. For the binding of uranyl, several mechanisms were suggested: (a) uranyl ions binding through direct interaction of U with both the N and O compounds of the oxime group (C=N-OH, after hydroxyl group deprotonation), (b) uranyl ions binding through four different O on groups of OH derived from the vicinal function of amidoxime, (c) an interaction with N from the group of amine and from the group of oxime, and (d) a “ring structure” formation through interactions of ions of the uranyl model pollutant with adjacent groups of amidoximes with simultaneous bonds between the cation of uranyl and O from groups of oxime, and also N from groups of amine. The interaction of the multidentate ligand with species of uranyl is facilitated in the case where free species of uranyl predominate in the solution [16].

In the study of Mohammad G.Mahfouz et al., the adsorption of U(VI) from the aqueous solution, using magnetic nano-based particles of diethylenetriamine-functionalized CS, was investigated. In the study of Ritcey and Ashbrook, the solvent extraction of ions derived from uranyl, using solutions from sulfuric acid by extractants of the amine type, is reported. The concentration of sulfate and the value of pH control the speciation of metal via the favorable formation of anionic and neutral species. In the presence of high HSO_4_^−^ concentrations (acidic solutions), the ions of uranyl may react and form uranium bisulfate, which can be mentioned as poorly extractable. However, in the case of high values of pH (sulfate excess), the functions of amine are unable to be presented under their salt form, and consequently cannot extract the species of anionic uranium sulfate. Thus, it can be concluded that, in order to extract uranyl from the aqueous solution, an intermediary range of pH is the proper pH. Based on the aforementioned mechanism, the uranyl ions sorption in acid media (sulfuric) can be described according to the following Equations (4), (5), (6a–c), (7a–c), and (8a–c) [15]:

UO_2_^2+^ + 2SO_4_^2−^ → UO_2_ (SO_4_)_2_^2−^ (4)

UO_2_ (SO_4_)_2_^2−^ + SO_4_^2−^ → UO_2_ (SO_4_)_3_^4−^ (5)

In addition, the mechanism of sorption of uranium ions onto tertiary amines is mainly based on (a) the metal ions ability to form species with an anionic charge in the aqueous phase, and (b) their capacity to be bound by groups of amines through a process of anion exchange. The amine compound, in the first step, should be converted to the appropriate polar ion pair or amine salt (Equation (6a)). In the second step, according to Equations (7a) and (8a), this makes the exchange of metal ions with the co-anion possible, and is given by:

2R_3_N + H_2_SO_4_ ↔ (R_3_NH^+^)_2_ {SO_4_}^2−^ (6a)

2R_2_NH + H_2_SO_4_ ↔ (R_2_NH^+^_2_)_2_ {SO_4_}^2−^ (6b)

2RNH_2_ + H_2_SO_4_ ↔ (R_2_NH^+^_3_)_2_ {SO_4_}^2−^ (6c)

2(R_3_NH^+^)_2_ {SO_4_}^2−^ + UO_2_(SO_4_)_3_^4−^ ↔ (R_3_NH^+^)_4_ {UO_2_(SO_4_)_3_}^4−^ + 2SO_4_^2−^ (7a)

2(R_2_NH^+^_2_)_2_ {SO_4_}^2−^ + UO_2_ (SO_4_)_3_^4−^ ↔ (R_2_NH^+^_2_)_4_ {UO_2_ (SO_4_)_3_}^4−^ + 2SO_4_^2−^ (7b)

2(RNH^+^_3_)_2_ {SO_4_}^2−^ + UO_2_ (SO_4_)_3_^4−^ ↔ (RNH^+^_3_)_4_ {UO_2_ (SO_4_)_3_}^4−^+2SO_4_^2−^ (7c)

However, by analogy with tertiary amines, similar reactions are expected to happen with secondary or even primary amines like Equations (6b,c); (7b,c) and (8b,c)

2(R_3_NH^+^)_2_ {SO_4_}^2−^ + UO_2_ (SO_4_)_2_^2−^ ↔ (R_3_NH^+^)_2_ {UO_2_(SO_4_)_2_}^2−^+ SO_4_^2−^ (8a)

2(R_2_NH^+^_2_)_2_ {SO_4_}^2−^ + UO_2_ (SO_4_)_2_^2−^ ↔ (R_2_NH^+^_2_)_2_ {UO_2_ (SO_4_)_2_}^2−^+SO_4_^2−^ (8b)

2(RNH^+^_3_)_2_ {SO_4_}^2−^ + UO_2_ (SO_4_)_2_^2−^ ↔ (RNH^+^_3_)_2_ {UO_2_ (SO_4_)_2_}^2−^ + SO_4_^2−^ (8c)

Another extraction mechanism, for uranium from acidic media (sulfate), has been presented to operate in the case of tertiary amines; namely, the extraction of neutral species of uranium sulfate via an adduct-type mechanism [15]:

(R_3_NH^+^)_2_{SO_4_^2−^} + UO_2_ SO_4_ ↔ (R_3_NH^+^)_2_ {UO_2_ (SO_4_)_2_}^2−^ (9a)

And by analogy:

(R_2_NH^+^_2_)_2_ {SO_4_^2−^} + UO_2_SO_4_ ↔ (R_2_NH^+^_2_)_2_{UO_2_(SO_4_)_2_}^2−^ (9b)

(RNH^+^_3_)_2_ {SO_4_^2−^} + UO_2_SO_4_ ↔ (RNH^+^_3_)_2_ {UO_2_(SO_4_)_2_}^2−^ (9c)

**Figure 22:**
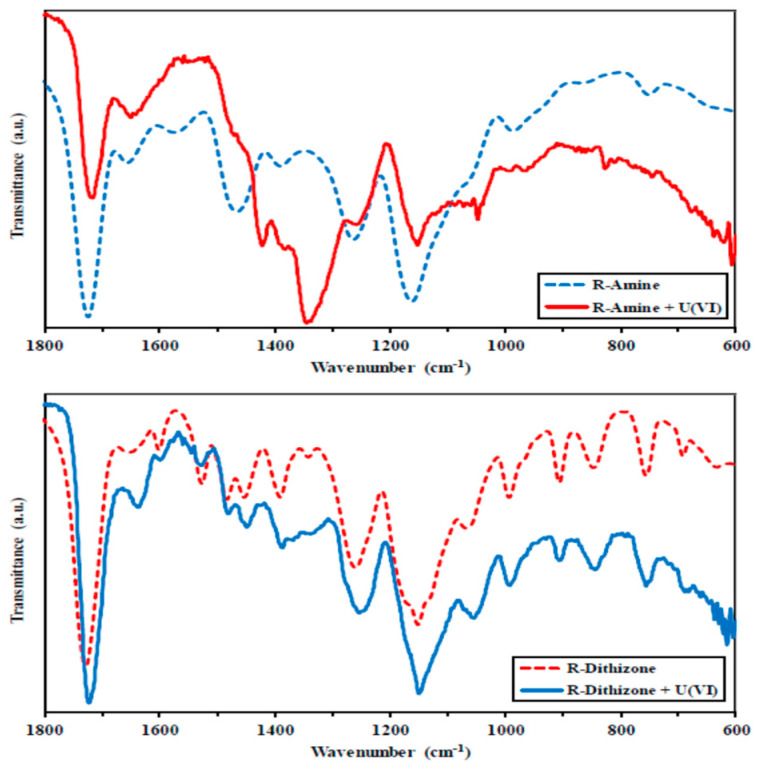
FTIR spectra of R-Amine and R-Dithizone composite nanoadsorbents (before and after adsorption of U(VI)). Reprinted from Ref [13]. Copyright 2021 with permission from Elsevier B.V.

Finally in the case of U(VI) adsorption, in the study of Elwakeel et al., the possible adsorption mechanism of U(VI) onto magnetic-GMA-chitosan, R-Amine, and R-Dithizone composite nanoadsorbent was tested. In Figure 22, it can clearly be observed that the capture of uranyl ions strongly affects the spectra derived from FTIR. In the case of R-Amine, the band at 1723 cm^−1^, corresponding to stretching in carboxylate (C=O), is shifted to 1716 cm^−1^, and free groups of carboxyl (resulting from the opening of the epoxy ring) may participate in the binding of the metal, or may also be attributed to the charge change in pH (controlled pH). The peak at 1567 cm^−1^, corresponding to the bending of N-H, disappears, which is a result that confirms the strong contribution of groups of amines in the removal of U(VI) metal ions. The peak at 1466 cm^−1^, corresponding to the stretching of C-N, is also considerably decreased, while two new bands appear at 1343 cm^−1^ and 1422 cm^−1^. In addition, another band that is affected by the adsorption of uranyl ions is the band of the carbohydrate ring at 1163 cm^−1^. 

However, for R-Dithizone, it can clearly be observed that the band at 1728 cm^−1^, corresponding to stretching in carboxylate (C=O), is shifted to 1721 cm^−1^. As aforementioned, this result may be attributed to the fact that the C=O groups are involved in the U(VI) uptake, or may also be attributed to the charge change in pH (controlled pH). The position and the intensity of some peaks of R-Dithizone are slightly changed. 

Furthermore, some significant changes (many small peaks appear) in the range of 1370–1315 cm^−1^ (poorly resolved zone) can clearly be observed. In addition, the band at 1257 cm^−1^ is widened (convoluted spectrum) and covers two poorly resolved contributions (at least). Moreover, the region at 1211–1078 cm^−1^ is also affected by the sorption of uranyl, the 1184 cm^−1^ and 1132 cm^−1^ shoulders are weakened, and the chemical environment of S = C-N is possibly affected by the binding of uranyl. This result is also confirmed by the band splitting at the peak of 1453 cm^−1^, corresponding to the stretching of S = C-N, with a band enlargement (new peak formation at 1448 cm^−1^). The other peaks (993 cm^−1^, 906 cm^−1^, 845 cm^−1^, 754 cm^−1^, and 690 cm^−1^) are slightly modified (some of the splits are attributed to the bands of uranyl nitrate). Indeed, it must be noted that the uranyl ions binding is frequently correlated with the forming of a signal at 917-912 cm^−1^, which corresponds to the O=U=O unit (asymmetric stretching vibration). Here, this band is “hidden” in the range of 970–900 cm^−1^ (poorly resolved region). A series of smalls bands at 1343 cm^−1^, 826 cm^−1^, and 745 cm^−1^ could be attributed to the ligand of nitrate attached to bound uranyl (−N=O symmetric bending, −N−O out of plane rocking, −N−O symmetric bending, respectively) [13].

**Figure 23:**
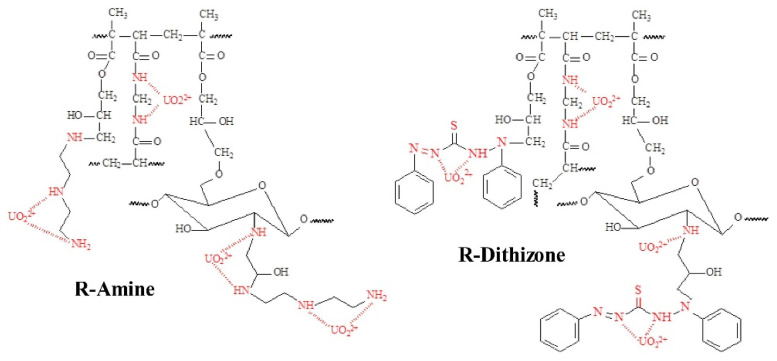
Interaction of R-Amine and R-Dithizone with U(VI) and tentative mechanisms. Reprint from Ref [13]. Copyright 2021 with permission from Elsevier B.V.

The adsorption of uranium onto R-Amine was achieved via interactions with the groups of amines, and with the possible COO- contribution due to the residual epoxy ring opening. In the case of R-Dithizone, in addition to the aforementioned functional groups, the change in the S=C-N chemical environment confirms that these groups (reactive) may also take part in the binding of uranyl (possibly bound as a form of nitrate). Figure 23 summarizes and depicts the tentative mechanisms for the bending of uranium onto R-Amine and R-Dithizone composite nanoadsorbents, respectively [13].

### 3.6. Adsorption Mechanisms for the Removal of Mercury from Aqueous Solution

**Figure 24:**
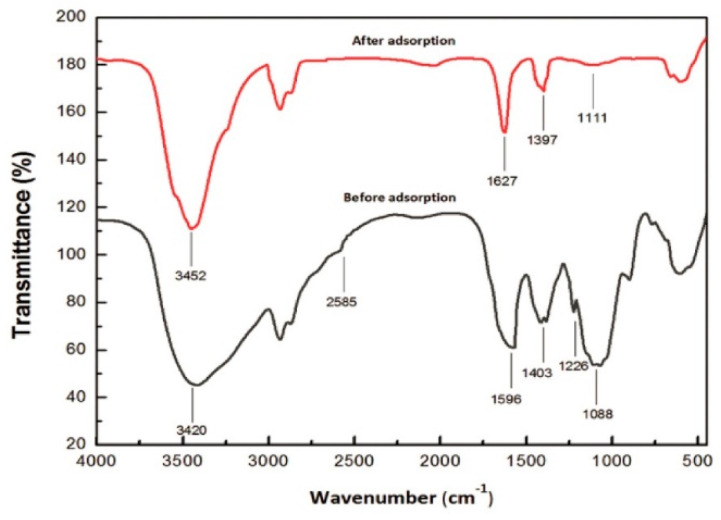
FTIR spectra derived from thiol-functionalized CS composite nanoadsorbent before and after Hg (II) adsorption. Reprinted from Ref [26]. Copyright 2019 with permission from Elsevier Ltd.

Below, some selected studies for the adsorption of Hg (II) onto chitosan composite nanoadsorbents are presented. In the study of Yasaman Nemati et al., the possible adsorption mechanism of Hg (II) onto thiol-functionalized CS NPs composite nanoadsorbent was investigated. In Figure 24, the FTIR spectra of microfluidic-fabricated thiol-functionalized CS NPs before and after Hg (II) adsorption is presented. Before the process of adsorption, two characteristic bands at 1596 cm^−1^ and 1403 cm^−1^ can clearly be observed, which peaked after the process of adsorption shifted to 1627 cm^−1^ and 1397 cm^−1^, respectively. This result is attributed to the formation of Hg (II) chelate with N atoms derived from amino groups. Furthermore, the strong band at 3420 cm^−1^ corresponds to -OH groups shifted to 3452 cm^−1^, which is a result that indicates that the -OH functional group positively influences the process of Hg (II) adsorption. However, the band at 1088 cm^−1^ changed to 1111 cm^−1^ and the intensity was also reduced, suggesting that the O atom in the groups of hydroxyl also participated in the process of Hg (II) adsorption. This result was in agreement with other research for the uptake of metal ions, such as Ngah and Fatinathan (2010), who recommended that the Cu (II) adsorption onto CS-TPP beads was possibly attributed to the mechanism of chelation involving the O (from hydroxyl groups), and N (from -NH_2_) atoms. In addition, another reduced band was at 2585 cm^−1^, suggesting that the groups of sulfur were also involved in the binding of the metal ion through the mechanisms of chelation. The sites of sulfur were reactive groups for the adsorption of metal ions because Hg (II) is a soft acid that shows an affinity for soft bases, such as sulfur atoms. Finally, the amount of groups of P=O at 1226 cm^−1^ was negligible after the adsorption of Hg (II) ions, suggesting that the phosphate groups contribute to the removal of Hg (II) [26].

**Figure 25:**
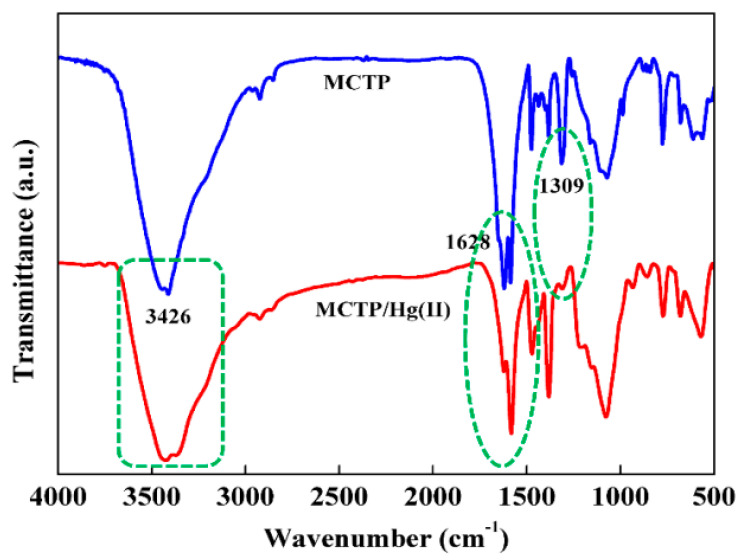
FTIR spectra of MCTP before and after the process of Hg (II) adsorption. Reprinted from Ref [25]. Copyright 2020 with permission from Elsevier B.V.

**Figure 26:**
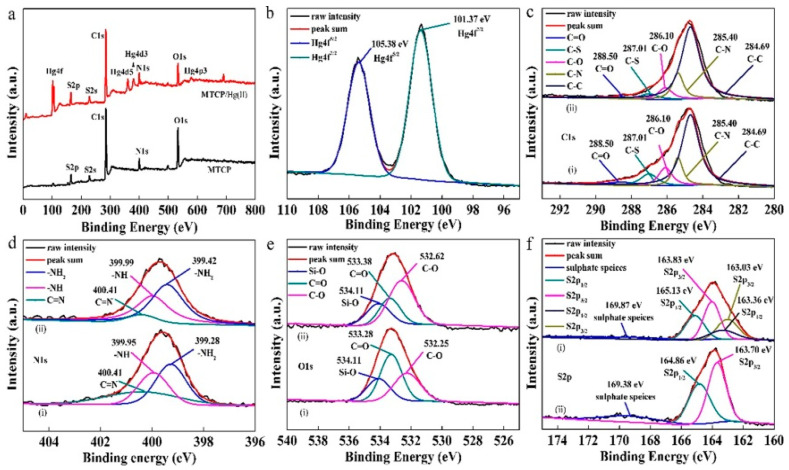
(**a**) Full survey XPS spectra; (**b**) high resolution Hg 4f; (**c**) C 1s; (**d**) N 1s; (**e**) O 1s; and (**f**) S2p spectra of MCTP composite nanoadsorbent; (i) MCTP and (ii) MCTP/Hg(II). Reprinted from Ref [25]. Copyright 2020 with permission from Elsevier B.V.

In another study by Yong Fu et al. the possible adsorption mechanism of Hg (II) onto the MCTP composite nanoadsorbent was investigated. In order to confirm the interaction between active groups and Hg (II) ions, the FTIR (Figure 25) and XPS spectra (Figure 26) were used for the analysis of MCTP before and after the adsorption of the Hg (II) model pollutant. The analysis with FTIR suggested that N and O groups might participate in the adsorption of Hg (II) due to the shifting of -NH_2_ and -OH, the stretching of the vibration peak, and the decrease in peak intensity of C-N and C=N. In addition, the analysis via XPS further confirmed the capture of Hg (II) onto the MCTP composite nanoadsorbent. Furthermore, the participation of groups with electron-rich atoms, such as -OH, -SH, -NH, and -NH_2_, in the complexation with ions of Hg (II), was indicated.

More specifically, according to the FTIR analysis of MCTP, the signals at 1628 cm^−1^ (C=N) and 1309 cm^−1^ (C-N) decreased, especially the C-N peak intensity. However, the band at 3426 cm^−1^ shifted to 3405 cm^−1^ (corresponding to -OH or -NH2 stretching vibration), which is a result that implies that the N or O groups might participate in the adsorption of Hg (II) [25].

The survey scans of MCTP before and after the process of Hg (II) capture, over the range of 0-800 eV, are shown in Figure 26a, where several new binding peaks of energy, including Hg4p3, Hg4d5, Hg4d3, and Hg4f could be observed in the spectrum of XPS of MCTP (after Hg (II) adsorption), indicating the Hg (II) presence in the MCTP composite nanoadsorbent. In Figure 26b, the high-resolution spectra derived from Hg4f is presented, where the doublet-peaks at an energy of binding of 105.38 eV and 101.37 eV (4.01 eV-binding energy gap), which correspond to Hg4f^5/2^ and Hg4f^7/2^, can clearly be observed. Moreover, the high-resolution XPS spectra of O 1s, N 1s, S 2p, and C 1s were analyzed in order to investigate the effect of S-, O-, N-, and C-contained functional groups on the adsorption of Hg (II), and these results are illustrated in Figure 26c–f. In Figure 26c, the deconvolution of spectrum derived from C 1s for the MCTP composite nanoadsorbent before and after the adsorption of Hg (II) is presented. The peaks at 288.50, 287.01, 286.10, 285.40, and 284.49 could be attributed to C=O, C-S, C-O, C-N, and C-C, where the intensity of all peaks (aforementioned) on C 1s, except the 284.69 eV, diminished after the adsorption of Hg(II). This result suggests that the S, O, and N atoms onto MCTP might be included in the Hg(II) adsorption. The deconvolution of N 1s depicted three major peaks at 339.28, 399.95, and 400.41 eV, in Figure 26d, which were assigned to -NH_2_, -NH, and C=N. After the process of Hg (II) adsorption, the energy of binding of -NH_2_ and -NH shifted to lower values (399.28–399.42, 399.95–399.99). It must be noted that the aforementioned results recommend that the groups of -NH and NH_2_ participate in the process of adsorption, which are results that are also confirmed via the FTIR analysis. Figure 26e presents the XPS spectra of the MCTP derived from O 1s that could be categorized into three peaks at 532.35, 533.28, and 534.11 eV, which could be attributed to C-O, C=O, and Si-O. Comparing the spectra of MCTP/Hg(II), it can be observed that the peaks of C-O (532.58) and C=O (533.38) present a shift (small displacement) to a high binding energy, which confirms that the Hg (II) model pollutant might have some interaction (weak) with adjacent -OH groups in the structure of the MCTP composite nanoadsorbent. As illustrated in Figure 26f(i), the S2p_3/2_, and S2p_1/2_ peaks appeared at 163.70 eV and 164.86 eV (free mercapto groups (-SH)), whereas the peaks that appeared at 169.38 eV could be attributed to species of sulphate SO_4_^2−^. After the process of Hg (II) adsorption, the two peaks of S2p appeared: one (S2p_1/2,3/2_ 163.03 eV, 163.36 eV) indicated that only part of the groups of -SH transformed to bonds of -S-Hg, whereas the other (S2p_1/2,3/2_ 163.83 eV, 165.13 eV) shifted to a high energy of binding when compared with the MCTP before Hg (II) adsorption, which is a result that indicates that the groups of sulfur also chelated with the model pollutant Hg (II) [25].

**Figure 27:**
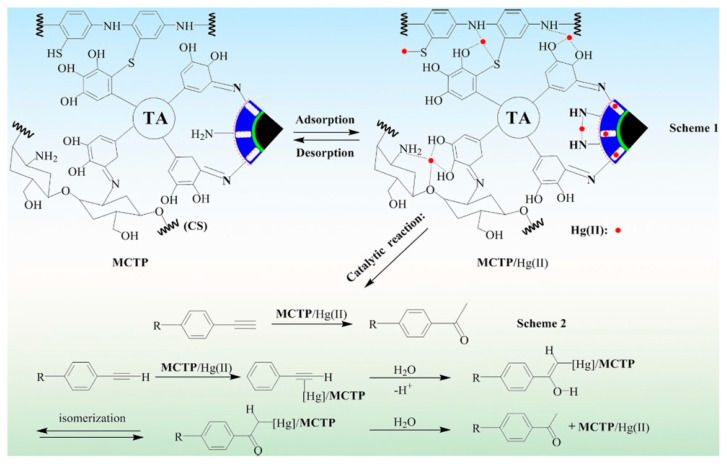
The mechanism of adsorption of Hg (II) onto MCTP and catalytic transformation mechanism. Reprinted from Ref [25]. Copyright 2020 with permission from Elsevier B.V.

According to the aforementioned results, the mechanism of adsorption could be assigned to the formation coordination bond with the Hg (II)-chelate or Hg (II) complex via multiple interactions, including the chelation reaction, coordination reaction, and electrostatic attraction. According to the Scheme 1 in Figure 27, the groups of -SH initially had an important role in the quick uptake of the Hg (II) model pollutant via the formation coordination bond with Hg (II). In addition, the chelate complexes of Hg (II) were formed through coordination bonds with -NH_2_ or -NH, and -SH, which was also dominant in the capture of the Hg (II) model pollutant. Moreover, the -OH, -NH_2_, -NH, and -SH functional groups also share their lone pair of electrons in order to capture the ions of Hg (II) via an electrostatic interaction, which, in turn, could form the complexes of -OH Hg (II), -NH_2_ Hg (II), -NH Hg (II), and -SH Hg (II). Finally, after the full occupation of available binding sites, the ions of Hg (II) might be both migrated from the surface of the MCTP composite nanoadsorbent into the nano-pores structure via the process of intraparticle diffusion, and adsorbed via the process of physical adsorption [25]. 

Finally, in the study of Amir Reza Sadrolhosseini et al., the possible adsorption mechanism of Hg(II) onto the PPy-Chi/NiFe_2_O_4_-NPs composite layer was investigated. In addition, the composite layer of PPy-Chi/NiFe_2_O_4_-NPs contained CS and NiFe_2_O_4_-NPs. The main operators that were employed in order to adsorb heavy metal ions were CS and the magnetic properties derived from NiFe_2_O_4_-NPs. The amino and hydroxyl groups of the CS, -NH_2_, and -OH, respectively, can adsorb ions derived from heavy metals, and the CS affinity corresponds to the heavy metal. Furthermore, the -NH_2_ group of CS is the primary group that is involved in the binding of metal ions, and it is widely acceptable that ions derived from heavy metals are immobilized onto CS via four amino groups in square-planar geometry.

NiFe_2_O_4_-NPs have a ferromagnetic behavior, and consequently can adsorb heavy metal ions with ferromagnetic properties (Ni, Fe, Co) more quickly and easily. However, the Hg (II) has diamagnetic properties, which results in the NiFe_2_O_4_-NPs not being able to adsorb it; however, the Hg (II) model pollutant can attach to the chain of CS [29].

## 4. Conclusions

The composite nanoadsorbents derived from the chitosan biopolymer are currently in their peak, due to their increased adsorption abilities, and many researchers worldwide focus on this biopolymer compound in their synthesis. Several CS composite adsorbents, with an enhanced adsorption capacity and superior resistance in extreme media conditions, have been produced via sundries methods, such as the grafting process and cross-linking reactions. This review study focused on uranium, mercury, and REEs removal from an aqueous solution with CS adsorbent composites, demonstrating the CS composites with the higher adsorption capacity. Specifically, in the case of the uranium compounds uptake from the aqueous solution, the adsorption capacity of FCCP was found to be 625 mg/g at pH 8, whereas, in the case of mercury compounds, the adsorption capacity was higher: 1126 mg/g at pH 5.25, using thiol-functionalized CS NPs. These values were higher when compared with other synthesized adsorbent chitosan materials. Furthermore, it was found that europium was removed more satisfactorily from the aqueous solution (its adsorption capacity was found to be 375.4, at pH 5) compared with other rare earth element compounds, by using magnetic chitosan microparticles grafted with malonitrile and amidoximated. However, positive results were also presented in the case of dysprosium, neodymium, and terbium rare earth element compounds removal from the aqueous solution, using a CA/CMC/Ni_0.2_Zn_0.2_Fe_2.6_O_4_ composite nanoadsorbent, where the adsorption capacity was found to be 114.74 mg/g, 73.37 mg/g, and 101.61 mg/g, respectively, at pH 5.5. It must be noted that, in general, the efficiency of the adsorption process depends on the molecules’ molecular weight (model pollutants), functional groups (adsorbent-adsorbate), degree of dilution in synthetic aqueous solution, pH effect, temperature of aqueous solution, techniques such as mechanical agitation, ultrasonic and/or microwave treatment, etc. It is concluded that the preparation of CS adsorbents is very effective for the removal of uranium, mercury, and REEs (La, Nd, Gd, Dy, Er, Tb etc.) compounds from aqueous solutions, and is a fruitful research area for further studies. The modification of CS with various functional groups, study of the effect of CS molecular weight on the adsorption process, or varying of the critical parameters (pH, temperature) during adsorption are fields for further studies. Furthermore, additional research can be conducted for the simultaneous removal of heavy metals and REEs from aqueous solutions for the improvement of the water’s quality. Along with the aforementioned benefits, CS adsorbents are an economically sustained alternate solution for water treatment applications. The effective removal of the elements is conducted with low cost procedures and reagents. Their synthesis is simple, whereas the required amounts of organic solvents for their preparation are negligible. Overall, in the next decade, CS adsorbents are expected to be further used for various types of adsorption applications.

## Figures and Tables

**Table 1:** Lists of adsorption isotherms (non-linear forms).

Isotherm	Non-Linear Form
Langmuir	$Q_{e}=\frac{Q_{m}K_{L}C_{e}}{1+K_{L}C_{e}}$
Freundlich	$Q_{e}=K_{F}{\left(C_{e}\right)}^{1/n}$
Langmuir-Freundlich	$Q_{e}=\frac{Q_{m}K_{LF}C_{e}{}^{1/b}}{1+K_{LF}C_{e}{}^{1/b}}$
Dubinin-Radushkevich	$Q_{e}=\left(Q_{s}\right)e^{-k_{DR}\varepsilon^{2}}$
Tempkin	$Q_{e}=\left(\frac{RT}{b_{T}}\right)ln\left(A_{T}C_{e}\right)$
Flory-Huggins	$\frac{\theta }{C_{0}}=K_{FH}{\left(1-\theta \right)}^{n_{FH}}$
Hill	$Q_{e}=\frac{Q_{S_{H}}C_{e}^{n_{H}}}{K_{D}+C_{e}^{n_{H}}}$
Redlich-Peterson	$Q_{e}=\frac{K_{R}C_{e}^{}}{1+a_{R}C_{e}^{g}}$
Sips	$Q_{e}=\frac{K_{S}C_{e}^{\beta_{S}}}{1+a_{S}C_{e}^{\beta_{S}}}$
Toth	$Q_{e}=\frac{K_{T}C_{e}^{}}{{\left(a_{T}+C_{e}\right)}^{1/t}}$
Koble-Corrigan	$Q_{e}=\frac{AC_{e}^{n}}{1+BC_{e}^{n}}$
Khan	$Q_{e}=\frac{Q_{s}b_{K}C_{e}^{}}{{\left(1+b_{K}C_{e}^{}\right)}^{a_{K}}}$
Radke-Prausnitz	$Q_{e}=\frac{a_{RP}r_{R}C_{e}^{\beta_{R}}}{a_{RP}+r_{R}C_{e}^{\beta_{R}-1}}$
BET	$Q_{e}=\frac{Q_{s}C_{BET}C_{e}}{\left(C_{s}-C_{e}\right)\left[1+\left(C_{BET}-1\right)\left(C_{e}/C_{s}\right)\right]}$
FHH	$ln\left(\frac{C_{e}}{C_{s}}\right)=-\frac{\alpha }{RT}{\left(\frac{Q_{s}}{Q_{e}d}\right)}^{r}$
MET	$Q_{e}=Q_{s}{\left(\frac{kC_{e}}{ln\left(C_{s}-C_{e}\right)}\right)}^{1/3}$

**Table 2:** Lists of kinetic equations used.

Isotherm	Equation Form
Pseudo-first order (non-linear)	$Q_{t}=Q_{e}\left(1-e^{-k_{1}t}\right)$
Pseudo-first order (linear)	$ln\left(Q_{e}-Q_{t}\right)=ln\left(Q_{e}\right)-k_{1}t\;\;$
Pseudo-second order (non-linear)	$Q_{t}=\frac{k_{2}Q_{e}{}^{2}t}{1+k_{2}Q_{e}t}$
Pseudo-second order (linear)	$\frac{t}{Q_{t}}=\frac{1}{k_{2}Q_{e}{}^{2}}+\left(\frac{1}{Q_{e}}\right)t$
Elovich	$Q_{t}=\frac{1}{\beta_{\mathrm{el}}}\ln\left(a\cdot \beta_{\mathrm{el}}\right)+\frac{1}{\beta_{\mathrm{el}}}ln\left(t\right)$
Intraparticle diffusion	$Q_{t}{=k}_{D}t^{1/2}$

**Table 3:** Selected studies for the adsorption of uranium from aqueous solutions, at 25 °C, using modified chitosan adsorbents. The optimum mathematical fitting of isotherm and kinetic models, derived from the experimental results after the adsorption process, are abbreviated with parenthesis.

Sorbent	pH	Model Pollutant	Isotherms	Kinetics	Q_max_ (mg/g)	References
Hydrothermal cross-linking CS-Fe_3_O_4_ (HCC-Fe_3_O_4_)	7	U(VI)	(L), F	PFO, (PSO)	263.1	[10]
TPP-crosslinked CS-Fe_3_O_4_	4	U(VI)	(L), F	Not presented	169.5	[1]
CS- Fe_3_O_4_ cross-linked with epichlorohydrin followed by grafting of triethylenetetramine	4	U(VI)	Not presented	Not presented	158.43	[18]
CS- Fe_3_O_4_ microparticles are functionalized by grafting a new hydrazide	5	U(VI)	L	(PFO), PSO	368.94	[19]
Glycine-grafted CS- Fe_3_O_4_ (HGly)	~6	U(VI)				[42]
Μagnetic-Momordica charantia leaf powder impregnated into chitosan (m-MCLPICS)	5	U(VI)	(L), F, D-R	PFO, (PSO)	250.7	[11]
2-phosphonobutane-1,2,4-tricarboxylic acid (PBTCA)-decorated chitosan-coated magnetic silica nanoparticle (CoFe_2_O_4_@SiO_2_@CS-PBTCA)	4	U(VI)	(L), F	PFO, (PSO), I-PD	105.26	[9]
Polyethylenimine-functionalized magnetic chitosan nanoparticles (MCN-PEI)	5	U(VI)	(L), F	PFO, (PSO)	134.6	[17]
Magnetic amidoxime functional chitosan (MAO-chitosan)	6	U(VI)	(L), F, T	PFO, (PSO), ELV, I-PD	117.65	[12]
Humic acid modified magnetic chitosan nano particles (HA-MCNP)	5–7	U(VI)	(L), F	PFO, (PSO)	47.9	[14]
Phosphate- and amide-functionalized magnetic CS-carboxymethylcellulose composite (FCCP)	8	U(VI)	(L), F, D-R	PFO, (PSO), I-PD	625	[20]
Epichlorohydrin-activated magnetic chitosan micro-particles (EPI-MG-CH)	4	U(VI)	(L), F, S	PFO, (PSO)	357	[16]
Magnetic nano-based particles of diethylenetriamine-functionalized CS	3.6	U(VI)	(L), F, D-R	PFO, (PSO), I-PD	177.93	[15]
Magnetic-GMA-chitosan, under mechanical agitation (210 rpm)	2.7	U(VI)	(L), F, (S)	Not presented	185.66	[13]
R-amine, under mechanical agitation (210 rpm)	5.7	U(VI)	L, F, (S)	(PFO), PSO, I-PD	557	[13]
R-Dithizone, under mechanical agitation (210 rpm)	5.7	U(VI)	L, F, (S)	PFO, PSO, IPD	423.7	[13]
R-Amine, under ultrasonic treatment (80 kHz)	5.7	U(VI)	L, F, (S)	PFO, (PSO), IPD	559.4	[13]
R-Dithizone, under ultrasonic treatment (80 kHz)	5.7	U(VI)	L, F, (S)	PFO, (PSO), IPD	583.2	[13]
R-Amine, under microwave treatment (2.45 GHz)	5.7	U(VI)	(L), F, (S)	PFO, (PSO), IPD	428.5	[13]
R-Dithizone, under microwave treatment (2.45 GHz)	5.7	U(VI)	L, F, (S)	PFO, (PSO), IPD	203	[13]

**Table 4:** Isotherm evaluation of U(VI) ions onto m-MCLPICS. Reprinted from Ref [11]. Copyright 2020 with permission from Elsevier B.V.

Isotherm	Parameters	Values
Langmuir	Q_max,cal_ (mg/g)	250.7
	K_L_ (L/mg)	0.036
	R^2^	0.9923
	χ^2^	34.5
Freundlich	K_F_ (mg/g)	52.6
	n	3.747
	R^2^	0.9752
	χ^2^	107.6
Dubinin-Radushkevich	Q_max,cal_ (mg/g)	216.1
	K	0.0271
	R^2^	0.5736
	χ^2^	816.5

**Table 5:** Evaluation of kinetic parameters for the adsorption of U(VI) onto m-MCLPICS. Reprinted from Ref [11]. Copyright 2020 with permission from Elsevier B.V.

Adsorbent	C_U(VI)_		PFO			PSO		
		q_e,exp_ (mg/g)	q_e,cal_ (mg/g)	k_1_ (L/min)	R^2^	q_e,cal_ (mg/g)	k_2_ (g/mg min)	R^2^
m-MCLPICS	20	60.2	51.2	0.1183	0.9784	59.06	0.0035	0.9967
	40	79.1	68.7	0.1127	0.9714	79.8	0.0026	0.9949
	60	120.4	101.1	0.1134	0.9874	118.9	0.0016	0.9983
	80	151.4	140.1	0.1183	0.9795	149.5	0.0014	0.9956

**Table 6:** Selected studies for the adsorption of mercury from aqueous solutions, at 25 °C, using modified chitosan adsorbents. The optimum mathematical fitting of isotherm and kinetic models, derived from the experimental results after the adsorption process, are abbreviated with parenthesis.

Sorbent	pH	Isotherms	Kinetics	Q_max_ (mg/g)	Ref.
Magnetic chitosan flakes-cross-linking EDTA-Na_2_ (MCFs-EDTA-Na_2_)	4.7	(L), F	PFO, (PSO), I-PD, ELV	495	[22]
Naked magnetic chitosan flakes coated Fe_3_O_4_ micro-particles (NMCFs)	4.6	(L), F	PFO, (PSO), I-PD, ELV	454	[22]
Thiol-functionalized CS NPs	5.25	L, F, T, D-R, (R-P), (R-P), (UT)		1126	[26]
Magnetic network polymer composite (MCTP)	3.5	(L), F	PFO, (PSO), I-PD	506.39	[25]
Chitosan/magnetite nanocomposite (M.Cs.NC.)	7	L, (F), T, D-R	FO, SO, PFO, (PSO)	125	[24]
Chitosan and p-sulfonato dansyl calix(4) arene composite (Fe3O4@Chitosan-pSDCalix)		(L), F		86.65	[21]
Chitosan-Iron(III) beads	4.5–5.0	(L), F, S	PFO, (PSO)	361.1	[23]
AT-MCS nano-biosorbent	7	(L), F	PFO, (PSO), I-PD	245.6	[27]
Polypyrrole-chitosan/nickel-ferrite nanoparticle composite layer (PPy-Chi/NiFe_2_O_4_ composite layer)	7.52–7.58	(L)	PFO		[29]
Glutamine modified chitosan magnetic composite microspheres (CS-Gln-MCM)	5	(L), F	PFO, (PSO), I-PD	199.23	[28]

**Table 7:** Selected studies for the adsorption of rare earth elements from aqueous solutions, at 25 °C, using modified chitosan adsorbents. The optimum mathematical fitting of isotherm and kinetic models, derived from the experimental results after the adsorption process, are abbreviated with parenthesis.

Sorbent	pH	Model Pollutant	Isotherms	Kinetics	Q_max_ (mg/g)	References
Fe_3_O_4_@ Chitosan nanocomposite	3	La(III)			105	[31]
Magnetic alginate-chitosan gel beads	2.8	La(III)	(L), F, D-R	PFO, (PSO)	97.1	[32]
Chitosan-manganese-ferrite magnetic beads	4	Nd(III)	L, F, S	PFO, PSO, ELV, I-PD	44.29	[33]
Magnetic calcium alginate/carboxymethyl chitosan/Ni_0.2_Zn_0.2_Fe_2.6_O_4_ (CA/CMC/Ni_0.2_Zn_0.2_Fe_2.6_O_4_)	5.5	Nd(III)	(L), F	PFO, (PSO), I-PD	73.37	[35]
Carboxymethyl chitosan/poly(pyrimidine-thiophene amide)/Ni_0.2_Zn_0.2_Fe_2.6_O_4_ (CMC/P(PTA)/Ni_0.2_Zn_0.2_Fe_2.6_O_4_)	5.5	Nd(III)	L, (F)	PFO, (PSO), (I-PD)	39.82	[37]
Fe_3_O_4_-octadecyltriethoxysilane (C_18_)-chitosan diethylenetriamine (DETA) composite	7	Nd(III)	(L), F, D-R	PFO, (PSO), I-PD	27.1	[38]
Calcium alginate/carboxymethyl chitosan/Ni_0.2_Zn_0.2_Fe_2.6_O_4_	5.5	Nd(III)	L, (F)	PFO, (PSO), I-PD	22.70	[36]
Diethylenetriaminepentaacetic acid (DTPA)-functionalized chitosan/magnetite (Fe_3_O_4_/NH_2_) nanocomposite	2-6	Gd(III)	(L), F		94.21	[39]
Chitosan/magnetite (Fe_3_O_4_/NH_2_) nanocomposite	7.23	Gd(III)	L, (F)		93.44	[34]
Magnetic calcium alginate/carboxymethyl chitosan/Ni_0.2_Zn_0.2_Fe_2.6_O_4_ (CA/CMC/Ni_0.2_Zn_0.2_Fe_2.6_O_4_)	5.5	Dy(III)	L, (F)	PFO, (PSO), I-PD	114.74	[35]
Carboxymethyl chitosan/poly(pyrimidine-thiophene-amide)/Ni_0.2_Zn_0.2_Fe_2.6_O_4_ (CMC/P(PTA)/Ni_0.2_Zn_0.2_Fe_2.6_O_4_)	5.5	Dy(III)	L, (F)	PFO, (PSO), (I-PD)	48.23	[37]
Fe_3_O_4_-octadecyltriethoxysilane (C_18_)-chitosan diethylenetriamine (DETA) composite	7	Dy(III)	(L), F, D-R	PFO, (PSO), I-PD	28.3	[38]
Calcium alginate/carboxymethyl chitosan/Ni_0.2_Zn_0.2_Fe_2.6_O_4_	5.5	Dy(III)	L, (F)	PFO, (PSO), I-PD	25.54	[36]
PCM-CS (Refers PCM-Chit)	5	Er(III)	(L), F	PFO, (PSO)	124.95	[40]
Fe_3_O_4_-octadecyltriethoxysilane (C_18_)-chitosan diethylenetriamine (DETA) composite	7	Er(III)	(L), F, D-R	PFO, (PSO), I-PD	30.6	[38]
Magnetic calcium alginate/carboxymethyl chitosan/Ni_0.2_Zn_0.2_Fe_2.6_O_4_ (CA/CMC/Ni_0.2_Zn_0.2_Fe_2.6_O_4_)	5.5	Tb(III)	L, (F)	PFO, (PSO), I-PD	101.61	[35]
Carboxymethyl chitosan/poly(pyrimidine-thiophene-amide)/Ni_0.2_Zn_0.2_Fe_2.6_O_4_ (CMC/P(PTA)/Ni_0.2_Zn_0.2_Fe_2.6_O_4_)	5.5	Tb(III)	L, (F)	PFO, (PSO), (I-PD)	50.32	[37]
Calcium alginate/carboxymethyl chitosan/Ni_0.2_Zn_0.2_Fe_2.6_O_4_	5.5	Tb(III)	L, (F)	PFO, (PSO), I-PD	24.00	[36]
Magnetic chitosan microparticles grafted with malonitrile and amidoximated	5	Eu(III)	(L), F, S	PFO, (PSO)	375.4	[16]

**Table 8:** Correlation coefficients and constants of L and F isotherm models for adsorption of Dy(III), Tb(III), and Nd(III) ions on CA/CMC/Ni_0.2_Zn_0.2_Fe_2.6_O_4_ composite adsorbent. Reprinted from Ref [35]. Copyright 2020 with permission from Elsevier B.V.

Models	Parameters	Dy(III)	Tb(III)	Nd(III)
Langmuir	b (L/mg)	0.546	0.8	1.075
	Q_max,cal_ (mg/g)	100.20	88.61	73.37
	R_L_	0.006	0.004	0.003
	R^2^	0.8212	0.7163	0.9703
	χ^2^	144.07	157.16	6.04
Freundlich	k (mg^1−1/n^ L^1/n^/g)	46.65	46.31	46.80
	n	5.93	6.83	10.31
	R^2^	0.9751	0.9905	0.8364
	χ^2^	20	5.23	33.32

**Table 9:** Correlation coefficients and constants of L and F isotherm models for adsorption of Dy(III), Tb(III), and Nd(III) ions on CMC/P(PTA)/Ni_0.2_Zn_0.2_Fe_2.6_O_4_ composite adsorbent. Reprinted from Ref [37]. Copyright 2020 with permission from Elsevier B.V.

Models	Parameters	Dy(III)	Tb(III)	Nd(III)
Langmuir	b (L/mg)	6.391	1.51	3.28
	Q_max,cal_ (mg/g)	43.76	42.87	36.59
	R_L_	0.0005	0.0022	0.001
	R^2^	0.5771	0.5398	0.5679
	χ^2^	47.31	43.21	16.7
Freundlich	k (mg^1−1/n^ L^1/n^/g)	26.13	23.7	24.23
	1/n	0.12	0.13	0.09
	R^2^	0.9632	0.9482	0.9709
	χ^2^	4.12	4.86	1.12

**Table 10:** Kinetic constants for adsorption of Dy(III), Tb(III), and Nd(III) ions on CA/CMC/Ni_0.2_Zn_0.2_Fe_2.6_O_4_ composite adsorbent, using PFO, PSO, and I-PD kinetic models. Reprinted from Ref [35]. Copyright 2020 with permission from Elsevier B.V.

Models	Parameters	Dy(III)	Tb(III)	Nd(III)
PFO	k_1_ (1/min)	0.269	0.271	0.279
	q_e,cal._ (mg/g)	46.54	46.05	45.91
	R^2^	0.9099	0.8712	0.8506
	χ^2^	5.34	7.29	7.91
PSO	κ_2_ (g/mg min)	0.00899	0.00916	0.00967
	q_e,cal._ (mg/g)	49.72	49.21	48.95
	h (mg/g min)	22.22	22.18	23.17
	R^2^	0.9956	0.9893	0.9831
	χ^2^	0.26	0.60	0.89
I-PD	k_i_ (1/min)	11.76	11.73	11.33
	R^2^	0.8723	0.9091	0.9063
	χ^2^	7.57	5.14	4.96

**Table 11:** Kinetic constants for adsorption of Dy(III), Tb(III), and Nd(III) ions on CMC/P(PTA)/Ni_0.2_Zn_0.2_Fe_2.6_O_4_ composite adsorbent, using PFO, PSO, and I-PD kinetic models. Reprinted from Ref [37]. Copyright 2020 with permission from Elsevier B.V.

Models	Parameters	Dy(III)	Tb(III)	Nd(III)
PFO	k_1_ (1/min)	0.126	0.132	0.116
	q_e,cal._ (mg/g)	32.71	32.29	31.15
	R^2^	0.8803	0.8538	0.8831
	χ^2^	7.52	8.77	7.24
PSO	k_2_ (g/mg min)	0.0047	0.0049	0.0043
	q_e,cal._ (mg/g)	36.27	35.80	34.85
	h (mg/g min)	6.18	6.28	5.22
	R^2^	0.9686	0.9564	0.9629
	χ^2^	1.97	2.61	2.3
I-PD	k_i_ (1/min)	11.2	11.02	11.16
	R^2^	0.9676	0.9782	0.9737
	χ^2^	2.03	1.31	1.63

## Data Availability

Not applicable.

## References

[1] Zhou L., Jia Y., Peng J., Liu Z., Al-Zaini E. (2014). Competitive adsorption of uranium(VI) and thorium(IV) ions from aqueous solution using triphosphate-crosslinked magnetic chitosan resins. J. Radioanal. Nucl. Chem..

[2] Vakili M., Deng S., Cagnetta G., Wang W., Meng P., Liu D., Yu G. (2019). Regeneration of chitosan-based adsorbents used in heavy metal adsorption: A review. Sep. Purif. Technol..

[3] da Silva Alves D.C., Healy B., Pinto L.A.d.A., Cadaval T.R.S.A., Breslin C.B. (2021). Recent Developments in Chitosan-Based Adsorbents for the Removal of Pollutants from Aqueous Environments. Molecules.

[4] Joshiba G.J., Kumar P.S., Christopher F.C., Govindaraj B.B. (2019). Insights of CMNPs in water pollution control. IET Nanobiotechnol..

[5] Ahmad M., Ahmed S., Swami B., Ikram S. (2015). Adsorption of heavy metal ions: Role of chitosan and cellulose for water treatment. Int. J. Pharmacogn..

[6] Fu F., Wang Q. (2011). Removal of heavy metal ions from wastewaters: A review. J. Environ. Manag..

[7] Zhou L., Ouyang J., Shehzad H., Le Z., Li Z., Adesina A. (2018). Adsorption of U(VI) onto the carboxymethylated chitosan/Na-bentonite membranes: Kinetic, isothermic and thermodynamic studies. J. Radioanal. Nucl. Chem..

[8] Zhao F., Sillanpää M., Sillanpää M. (2020). Chapter 3—Cross-linked chitosan and β-cyclodextrin as functional adsorbents in water treatment. Advanced Water Treatment.

[9] Huang Y., Zheng H., Li H., Zhao C., Zhao R., Li S. (2020). Highly selective uranium adsorption on 2-phosphonobutane-1,2,4-tricarboxylic acid-decorated chitosan-coated magnetic silica nanoparticles. Chem. Eng. J..

[10] Yu S.-L., Dai Y., Cao X.-H., Zhang Z.-B., Liu Y.-h., Ma H.-J., Xiao S.-J., Lai Z.-J., Chen H.-J., Zheng Z.-Y. (2016). Adsorption of uranium(VI) from aqueous solution using a novel magnetic hydrothermal cross-linking chitosan. J. Radioanal. Nucl. Chem..

[11] Yuvaraja G., Su M., Chen D.-Y., Pang Y., Kong L.-J., Subbaiah M.V., Wen J.-C., Reddy G.M. (2020). Impregnation of magnetic—Momordica charantia leaf powder into chitosan for the removal of U(VI) from aqueous and polluted wastewater. Int. J. Biol. Macromol..

[12] Zhuang S., Cheng R., Kang M., Wang J. (2018). Kinetic and equilibrium of U(Ⅵ) adsorption onto magnetic amidoxime-functionalized chitosan beads. J. Clean. Prod..

[13] Elwakeel K.Z., Hamza M.F., Guibal E. (2021). Effect of agitation mode (mechanical, ultrasound and microwave) on uranium sorption using amine- and dithizone-functionalized magnetic chitosan hybrid materials. Chem. Eng. J..

[14] Basu H., Saha S., Pimple M.V., Singhal R.K. (2019). Novel hybrid material humic acid impregnated magnetic chitosan nano particles for decontamination of uranium from aquatic environment. J. Environ. Chem. Eng..

[15] Mahfouz M.G., Galhoum A.A., Gomaa N.A., Abdel-Rehem S.S., Atia A.A., Vincent T., Guibal E. (2015). Uranium extraction using magnetic nano-based particles of diethylenetriamine-functionalized chitosan: Equilibrium and kinetic studies. Chem. Eng. J..

[16] Hamza M.F., Roux J.-C., Guibal E. (2018). Uranium and europium sorption on amidoxime-functionalized magnetic chitosan micro-particles. Chem. Eng. J..

[17] Sun G., Zhou L., Tang X., Le Z., Liu Z., Huang G. (2020). In situ formed magnetic chitosan nanoparticles functionalized with polyethylenimine for effective U(VI) sorption. J. Radioanal. Nucl. Chem..

[18] Tolba A.A. (2020). Evaluation of uranium adsorption using magnetic-polyamine chitosan from sulfate leach liquor of sela ore material, South Eastern Desert, Egypt. Egypt. J. Chem..

[19] Hamza M.F., Wei Y., Benettayeb A., Wang X., Guibal E. (2020). Efficient removal of uranium, cadmium and mercury from aqueous solutions using grafted hydrazide-micro-magnetite chitosan derivative. J. Mater. Sci..

[20] Guo X., Chen R., Liu Q., Liu J., Zhang H., Yu J., Li R., Zhang M., Wang J. (2018). Superhydrophilic phosphate and amide functionalized magnetic adsorbent: A new combination of anti-biofouling and uranium extraction from seawater. Environ. Sci. Nano.

[21] Bhatti A.A., Oguz M., Yilmaz M. (2018). One-pot synthesis of Fe_3_O_4_@Chitosan-pSDCalix hybrid nanomaterial for the detection and removal of Hg^2+^ ion from aqueous media. Appl. Surf. Sci..

[22] Nacer F. (2018). Comparative study of mercury(II) species removal onto naked and modified magnetic chitosan flakes coated ethylenediaminetetraacetic-disodium: Kinetic and thermodynamic modeling. Environ. Sci. Pollut. Res..

[23] Lapo B., Demey H., Zapata J., Romero C., Sastre A.M. (2018). Sorption of Hg(II) and Pb(II) Ions on Chitosan-Iron(III) from Aqueous Solutions: Single and Binary Systems. Polymers.

[24] Morsi R.E., Al-Sabagh A.M., Moustafa Y.M., ElKholy S.G., Sayed M.S. (2018). Polythiophene modified chitosan/magnetite nanocomposites for heavy metals and selective mercury removal. Egypt. J. Pet..

[25] Fu Y., Sun Y., Zheng Y., Jiang J., Yang C., Wang J., Hu J. (2021). New network polymer functionalized magnetic-mesoporous nanoparticle for rapid adsorption of Hg(II) and sequential efficient reutilization as a catalyst. Sep. Purif. Technol..

[26] Nemati Y., Zahedi P., Baghdadi M., Ramezani S. (2019). Microfluidics combined with ionic gelation method for production of nanoparticles based on thiol-functionalized chitosan to adsorb Hg (II) from aqueous solutions. J. Environ. Manag..

[27] Hou C., Zhao D., Zhang S., Wang Y. (2018). Highly selective adsorption of Hg(II) by the monodisperse magnetic functional chitosan nano-biosorbent. Colloid Polym. Sci..

[28] Tao X., Li K., Yan H., Yang H., Li A. (2016). Simultaneous removal of acid green 25 and mercury ions from aqueous solutions using glutamine modified chitosan magnetic composite microspheres. Environ. Pollut..

[29] Sadrolhosseini A.R., Naseri M., Rashid S.A. (2017). Polypyrrole-chitosan/nickel-ferrite nanoparticle composite layer for detecting heavy metal ions using surface plasmon resonance technique. Opt. Laser Technol..

[30] Ramasamy D.L., Puhakka V., Iftekhar S., Wojtuś A., Repo E., ben hammouda S., Iakovleva E., Sillanpää M. (2018). N-and O-ligand doped mesoporous silica-chitosan hybrid beads for the efficient, sustainable and selective recovery of rare earth elements (REE) from acid mine drainage (AMD): Understanding the significance of physical modification and conditioning of the polymer. J. Hazard. Mater..

[31] Rajabi N., Masrournia M., Abedi M. (2019). Measuring and Pre-concentration of Lanthanum Using Fe_3_O_4_@Chitosan Nanocomposite with Solid-phase Microextraction for ICP-OES Determination. Arab. J. Sci. Eng..

[32] Wu D., Zhang L., Wang L., Zhu B., Fan L. (2011). Adsorption of lanthanum by magnetic alginate-chitosan gel beads. J. Chem. Technol. Biotechnol..

[33] Durán S.V., Lapo B., Meneses M., Sastre A.M. (2020). Recovery of Neodymium (III) from Aqueous Phase by Chitosan-Manganese-Ferrite Magnetic Beads. Nanomaterials.

[34] Pylypchuk I., Kołodyńska D., Kozioł M., Gorbyk P. (2016). Gd-DTPA Adsorption on Chitosan/Magnetite Nanocomposites. Nanoscale Res. Lett..

[35] Javadian H., Ruiz M., Saleh T.A., Sastre A.M. (2020). Ca-alginate/carboxymethyl chitosan/Ni_0.2_Zn_0.2_Fe_2.6_O_4_ magnetic bionanocomposite: Synthesis, characterization and application for single adsorption of Nd^+3^, Tb^+3^, and Dy^+3^ rare earth elements from aqueous media. J. Mol. Liq..

[36] Javadian H., Ruiz M., Sastre A.M. (2020). Response surface methodology based on central composite design for simultaneous adsorption of rare earth elements using nanoporous calcium alginate/carboxymethyl chitosan microbiocomposite powder containing Ni0.2Zn0.2Fe2.6O4 magnetic nanoparticles: Batch and column studies. Int. J. Biol. Macromol..

[37] Javadian H., Ruiz M., Taghavi M., Sastre A.M. (2020). Synthesis of magnetic CMC bionanocomposite containing a novel biodegradable nanoporous polyamide selectively synthesized in ionic liquid as green media: Investigation on Nd^+3^, Tb^+3^, and Dy^+3^ rare earth elements adsorption. J. Mol. Liq..

[38] Liu E., Zheng X., Xu X., Zhang F., Liu E., Wang Y., Li C., Yan Y. (2017). Preparation of diethylenetriamine-modified magnetic chitosan nanoparticles for adsorption of rare-earth metal ions. New J. Chem..

[39] Pylypchuk I., Kołodyńska D., Gorbyk P. (2017). Gd(III) Adsorption on the Dtpa-Functionalized Chitosan/Magnetite Nanocomposites. Sep. Sci. Technol..

[40] Abd El-Magied M.O., Galhoum A.A., Atia A.A., Tolba A.A., Maize M.S., Vincent T., Guibal E. (2017). Cellulose and chitosan derivatives for enhanced sorption of erbium(III). Colloids Surf. A Physicochem. Eng. Asp..

[41] Kyzas G.Z., Matis K.A. (2015). Nanoadsorbents for pollutants removal: A review. J. Mol. Liq..

[42] Hamza M.F., Ahmed F.Y., El-Aassy I., Fouda A., Guibal E. (2018). Groundwater Purification in a Polymetallic Mining Area (SW Sinai, Egypt) Using Functionalized Magnetic Chitosan Particles. Water Air Soil Pollut..

